# High-throughput structures of protein–ligand complexes at room temperature using serial femtosecond crystallography

**DOI:** 10.1107/S2052252519011655

**Published:** 2019-10-10

**Authors:** Tadeo Moreno-Chicano, Ali Ebrahim, Danny Axford, Martin V. Appleby, John H. Beale, Amanda K. Chaplin, Helen M. E. Duyvesteyn, Reza A. Ghiladi, Shigeki Owada, Darren A. Sherrell, Richard W. Strange, Hiroshi Sugimoto, Kensuke Tono, Jonathan A. R. Worrall, Robin L. Owen, Michael A. Hough

**Affiliations:** a School of Life Sciences, University of Essex, Wivenhoe Park, Colchester CO4 3SQ, England; b Diamond Light Source, Harwell Science and Innovation Campus, Didcot OX11 0DE, England; c Division of Structural Biology (STRUBI), University of Oxford, The Henry Wellcome Building for Genomic Medicine, Roosevelt Drive, Oxford OX3 7BN, England; d Department of Chemistry, North Carolina State University, Raleigh, NC 27695-8204, USA; e RIKEN SPring-8 Center, 1-1-1 Kouto, Sayo, Hyogo 679-5148, Japan; f Japan Synchrotron Radiation Research Institute, 1-1-1 Kouto, Sayo, Hyogo 679-5198, Japan

**Keywords:** serial femtosecond crystallography, ligand binding, high throughput, X-ray crystallography, damage-free structures, X-ray free-electron lasers

## Abstract

High-throughput X-ray crystal structures of protein–ligand complexes are critical to pharmaceutical drug development. However, cryocooling of crystals and X-ray radiation damage may distort the observed ligand binding. Serial femtosecond crystallography (SFX) using X-ray free-electron lasers (XFELs) can produce radiation-damage-free room-temperature structures. Ligand-binding studies using SFX have received only modest attention, partly owing to limited beamtime availability and the large quantity of sample that is required per structure determination. Here, a high-throughput approach to determine room-temperature damage-free structures with excellent sample and time efficiency is demonstrated, allowing complexes to be characterized rapidly and without prohibitive sample requirements. This yields high-quality difference density maps allowing unambiguous ligand placement. Crucially, it is demonstrated that ligands similar in size or smaller than those used in fragment-based drug design may be clearly identified in data sets obtained from <1000 diffraction images. This efficiency in both sample and XFEL beamtime opens the door to true high-throughput screening of protein–ligand complexes using SFX.

## Abstract 2

The ability to rapidly obtain structures of protein–ligand complexes using X-ray crystallography is central to drug discovery, but the typical cryocooling of samples and the effects of the X-ray beam may distort the observed ligand binding. X-ray free-electron lasers (XFELs) have the promise to solve these issues, but methods to rapidly produce structures of protein–ligand complexes at XFELs have not yet been realized. Here, an efficient solution using high-throughput, fixed-target serial femtosecond crystallography at an XFEL is demonstrated.

## 1. Introduction   

The accurate determination of the structures of protein–ligand complexes is essential for drug discovery, enzymology and biotechnology. Developments in the automation of protein crystallization, ligand soaking, harvesting, structure determination, ligand modelling and structural refinement have allowed the high-throughput screening of soaked crystals at synchrotron X-ray beamlines (Collins *et al.*, 2018 ▸; Pearce, Krojer, Bradley *et al.*, 2017 ▸; Pearce, Krojer & von Delft, 2017 ▸). For important classes of proteins, the binding of ligands may be affected by X-ray-driven changes either in the oxidation state of redox centres within the protein or to amino-acid side chains involved in protein–ligand interactions. In these cases, there is a premium on structure determination using low-dose methods. Prime examples of this are heme enzymes, where the iron centre in resting iron(III) and high-valent iron(IV) states is exquisitely prone to reduction by solvated photoelectrons generated by the interaction of synchrotron X-rays with solvent in the crystal (see, for example, Beitlich *et al.*, 2007 ▸; Kekilli *et al.*, 2017 ▸). Heme enzymes, such as the cytochrome P450s, are involved in the metabolism/breakdown of approximately 90% of small-molecule drugs, and are more generally themselves drug targets in yeast, fungi and tuberculosis infections (McLean & Munro, 2017 ▸; Guengerich *et al.*, 2016 ▸; Rendic & Guengerich, 2015 ▸). Importantly, the determination of protein–ligand complexes at room temperature is likely to better reflect *in vivo* conditions than crystals cryogenically cooled to 100 K (for an interesting example, see Fischer *et al.*, 2015 ▸). Indeed, significant differences in binding have been observed at room temperature (RT) compared with 100 K (Keedy *et al.*, 2018 ▸). Furthermore, ligand soaking into microcrystals (1–20 µm) has the theoretical potential to be more effective than soaking into larger crystals (>50 µm) (McPherson, 2019 ▸). The distance that a ligand needs to penetrate into the crystal to reach its centre is proportionately shorter for smaller crystals, potentially leading to higher occupancy rates.

These issues in combination place a high value on protein–ligand complexes determined from microcrystals at RT that are free of observable effects of radiation damage. The only current approach that can deliver this is serial femtosecond crystallography (SFX) at X-ray free-electron lasers (XFELs; Schlichting, 2015 ▸) using short (<20 fs) X-ray pulses (Inoue *et al.*, 2016 ▸; Lomb *et al.*, 2011 ▸; Nass *et al.*, 2015 ▸; Nass, 2019 ▸). Ligand-binding studies using SFX have received little attention, largely owing to the scarcity of beamtime and high sample requirements in typical sample-delivery systems such as the gas dynamic virtual nozzle (GDVN) injectors (Schlichting, 2015 ▸). The drive to obtain damage-free, RT structures is balanced against the strong practical driver to minimize sample consumption per obtained structure, and the desire to collect data from multiple candidate ligands in a short time period.

A limited number of studies have sought to address the challenge of obtaining damage-free, RT crystal structures of protein–ligand complexes in a manner that is efficient both in sample and in data-collection time. An early study examined ligand binding to a P-type ATPase membrane protein in microcrystals delivered to the beam using a liquid microjet injector (Bublitz *et al.*, 2015 ▸). This work demonstrated the applicability of SFX to ligand-binding studies, showing that ligands could be clearly resolved even if the high-resolution data collected are weak and statistically poor. A more recent study (Naitow *et al.*, 2017 ▸) explored the feasibility of SFX ligand-binding studies using microcrystals of the model system thermolysin delivered by a high-viscosity water- or oil-based injector. The small-molecule ligand was readily resolved in electron-density maps, with clear differences in binding modes observed between the room-temperature SFX and 100 K synchrotron-radiation (SR) structures.

Here, we describe the rapid determination of protein–ligand complexes at RT. Microcrystals were mounted in silicon fixed targets or ‘chips’ at the SPring-8 Ångstrom Free Electron Laser (SACLA), Hyogo, Japan (Ishikawa *et al.*, 2012 ▸). The fixed-target sample-delivery approach minimizes sample consumption, provides high hit rates and allows multiple high-quality data sets to be measured in a very short time, an important advantage given the limited availability of XFEL beamtime. The chip system also allows rapid switching experiments in which crystals of different targets are soaked with different ligands. Moreover, the short time between soaking, chip loading and the completion of data collection reduces the need for long-term protein–ligand crystal stability that is required for a typical injector experiment. This also ensures that crystals are exposed to the soaked ligand for a similar length of time.

We have applied this approach to crystals of two heme peroxidase enzymes: a multifunctional dehaloperoxidase from the marine annelid *Amphitrite ornata* (DHP-B; Barrios *et al.*, 2014 ▸; Franzen *et al.*, 2012 ▸; McCombs, Moreno-Chicano, *et al.*, 2017 ▸; McCombs, Smirnova *et al.*, 2017 ▸) and a dye-decolourizing peroxidase (Sugano, 2009 ▸) of industrial relevance (Colpa *et al.*, 2014 ▸) from *Streptomyces lividans* (DtpAa). We also examine the challenging case of detecting nitrite binding to copper nitrite reductase from *Achromobacter cycloclastes* (*Ac*NiR; Horrell *et al.*, 2017 ▸), where the ligand displaces a water molecule bound in the active site. The enzyme and crystal systems used are of cubic (high), orthorhombic (medium) and monoclinic (low) symmetry space groups, as well as exhibiting full to partial ligand occupancies within the same crystallo­graphic asymmetric units. The complexes investigated include ligands directly binding to the heme, together with those occupying a binding pocket but not bound to the iron, with ligand sizes of 3–10 non-H atoms (Supplementary Fig. S1). We note that the typical molecular weight of the fragments used in fragment-based drug design is approximately 150–250 Da, with a typical size of 200 Da (Price *et al.*, 2017 ▸).

We explore the potential of this approach for rapid SFX screening of ligands/drug candidates, examining the minimum number of merged diffraction patterns required to reliably detect ligand binding and the future potential of this approach at current and planned XFEL beamlines. We assess several metrics for ligand fit to electron density with the data sets presented in the light of the recent debate around ligand validation (Smart *et al.*, 2018 ▸). Remarkably, data sets comprising of <1000 merged diffraction patterns allowed clear and unambiguous identification of ligand-binding modes, despite extremely poor merging and refinement statistics. The number of crystals required for complete data is lowered by the bandwidth of the XFEL beam. Our work thus demonstrates that high-throughput screening is eminently practicable using SFX, with modest requirements for sample quantity and experimental time.

## 2. Materials and methods   

### 2.1. Protein production and crystallization   

Dye-type peroxidase Aa (DtpAa) from *S. lividans* was expressed and purified as described previously (Ebrahim, Moreno-Chicano *et al.*, 2019 ▸). Crystals were grown in batch using a modification of the crystallization conditions used for growing large single crystals, consisting of 25%(*w*/*v*) PEG 1500 and 100 m*M* MIB buffer (Hampton Research, comprising MES, boric acid and imidazole pH 8). The final protein concentration in the batches ranged from 6.5 to 2.1 mg ml^−1^. Crystals grew in 1–2 days to approximate dimensions of 20–30 µm and were transported to SACLA at ambient temperature (the crystals were transported in hand luggage without cooling). Dehaloperoxidase B (DHP) from *A. ornata* was expressed and purified as described previously (McCombs, Moreno-Chicano *et al.*, 2017 ▸). Batch microcrystallization was used, mixing 30 mg ml^−1^ DHP in 20 m*M* MES pH 6.0 with 40%(*w*/*v*) PEG 4000, 200 m*M* ammonium sulfate in a 1:4 ratio in a total volume of 250–500 µl. DHP microcrystals grew in 3–5 days at 4°C to typical dimensions of 20–30 µm and were transported to SACLA at 4°C. 5-Bromoindole (5BR) and 2,4-dichlorophenol (DCP) (Sigma) were each dissolved in 100% DMSO and 20 µl of the resulting solution was added to a 200 µl crystal suspension to yield final ligand concentrations of 5 m*M* DCP and 50 m*M* 5BR. Microcrystals were soaked in batches for 3–5 min immediately prior to loading onto the silicon chip. *Ac*NiR microcrystals were grown as described previously (Ebrahim, Appleby *et al.*, 2019 ▸) and were soaked in 100 m*M* potassium nitrite for approximately 20 min prior to loading onto the chip.

### 2.2. Data collection and processing   

Microcrystals were loaded into fixed-target chips as described previously (Ebrahim, Appleby *et al.*, 2019 ▸; Oghbaey *et al.*, 2016 ▸). The chips were fabricated commercially (Southampton Nanofabrication Centre; https://www.southampton-nanofab.com) using a method based on that described previously (Oghbaey *et al.*, 2016 ▸). Typically, 100–200 µl of microcrystal suspension was loaded onto a chip containing 25 600 apertures and excess liquid was removed using a weak vacuum applied to the underside of the chip surface. For DHP microcrystals around 1.5 mg of protein was loaded in each chip, requiring around 4.5 mg for a complete data set (three or four chips), while *Ac*NiR microcrystals were loaded in quantities of around 2 mg for a complete data set (two chips at 1 mg per chip). In the case of DtpAa even less protein was needed: only 0.45–6.0 mg per chip and around 1.80 mg for a complete data set. SFX data were measured on SACLA (Ishikawa *et al.*, 2012 ▸) beamline BL2 EH3 with a photon energy of 10.0 keV, a repeat rate of 30 Hz and a pulse length of 10 fs. The beam, with a 1.25 × 1.34 µm spot size (FWHM) and a pulse energy of 289 µJ per pulse (pre-attenuation), was attenuated to 13% of full flux to minimize detector overloads. The SACLA beam was in SASE mode, with FWHM bandwidth ∼70 eV. The fixed-target chip was translated between X-ray pulses such that each crystal position was exposed only once, and the measurement of all 25 600 positions on a chip took 14 min. The hit rate during data collection was monitored using *Cheetah* (Barty *et al.*, 2014 ▸), while peak finding, indexing and merging of data were performed using *CrystFEL* v.0.6.4 (White *et al.*, 2016 ▸). Structures were refined using starting models of ligand-free structures from which water and other solvent molecules had been removed. Refinement was initially carried out in *REFMAC*5 (Murshudov *et al.*, 2011 ▸) within the *CCP*4 suite and completed in *PHENIX* (Adams *et al.*, 2010 ▸). All structures were validated using *MolProbity* (Williams *et al.*, 2018 ▸), the *JCSG Quality Control Check* server and tools within *PHENIX* (Adams *et al.*, 2010 ▸) and *Coot* (Emsley *et al.*, 2010 ▸).

To explore the limits of ligand identification in SFX data sets, randomly selected images from the indexed data (*.stream files from *CrystFEL*) formed data subsets with defined, variable numbers of images. These were scaled and merged in the same manner as the data sets containing all images and were used in refinement versus the model for the appropriate complex determined using all data, from which the ligand had been removed. OMIT maps were generated using torsion-based simulated-annealing refinement in *phenix.refine* (Adams *et al.*, 2010 ▸) in order to minimize model bias. As an additional validation step, selected subsets were refined against the structure of the native enzymes (where the ligands were not present) using the procedure described above.

### 2.3. Ligand modelling   

Ligands were initially modelled into the all-image data sets based on the *mF*_o_ − *DF*_c_ difference electron-density maps. In all cases, ligand density was unambiguous and ligands were modelled with near-full occupancy in one of the two subunits of the homodimeric enzymes (for DHP and DtpAa) or in the single subunit of *Ac*NiR in the crystallographic asymmetric unit. The ligands were straightforwardly located in an automated manner using the ‘Find Ligands’ feature of *Coot*. The second monomer in the DHP asymmetric unit contained a lower occupancy ligand (5BR) or very weak ligand density (DCP), while in DtpAa the second monomer did not show a bound exogenous ligand in the active site. Restraints for nonstandard ligands were produced using *ACEDRG* (Long *et al.*, 2017 ▸). For the data subsets, the data were refined by two parallel approaches to avoid model bias. Firstly, the data were refined against the ‘all-images’ structures, from which the ligands had been removed, using simulated annealing in *phenix.refine* to remove bias. As an additional test that bias was not present, selected structures were refined against the native, ligand-free structures of the enzymes and simulated-annealing (SA) OMIT difference maps were generated. The known position of the ligand from the ‘all-images’ models was then compared with the difference density map generated from that subset. The quality of the fit of modelled ligands to the electron-density maps was determined using *EDIAscorer* (Meyder *et al.*, 2017 ▸). The ‘Find Ligands’ feature of *Coot* was also used for each subset, in this case searching the *mF*_o_ − *DF*_c_ SA OMIT map for suitable hits.

*F*_o_ − *F*_o_ isomorphous difference maps between the DHP–5BR and DHP–DCP data sets were generated in *PHENIX* with the native ligand-free DHP structure (see below) used to phase the data sets (although near-identical results were generated if either of the above ligand-bound structures were used for phasing).

## 3. Results   

### 3.1. Determination of protein–ligand complex structures by SFX in a time- and sample-efficient manner   

SFX structures for each enzyme–ligand complex were determined from data measured from either two (*Ac*NiR), three (DHP–DCP) or four (DHP–5BR and DtpAa–imidazole) chips. This took approximately 14 min of data collection and ∼16 min of beamtime per chip (sample-change, hutch-search and alignment time are included). In each case, structure solution was by molecular replacement and the resolution and data quality were sufficient to clearly define essentially all main-chain and most side-chain atoms together with well defined networks of water molecules. The quality of the data sets and structures is given in Table 1 ▸. For each structure, clear positive difference density was evident for the ligands, which were unambiguously located. The chemical structures of the ligands used in this study are shown in Supplementary Fig. S1.

### 3.2. SFX structures of ligand-bound complexes   

In each DHP structure, clear *F*_o_ − *F*_c_ electron density was apparent in the heme pocket consistent with a high-occupancy bound ligand in one monomer of the dimer and a second lower occupancy binding site in the other. This difference in occupancy is consistent with previous single-crystal structures of DHP complexes with a range of different ligands in this space group (see, for example, McCombs, Moreno-Chicano *et al.*, 2017 ▸). DCP exhibited a binding site that was virtually identical to those previously observed for the guaiacol substrates 4-bromoguaiacol (PDB entry 6cke), 4-nitroguaiacol (PDB entry 6ch5) and 4-methoxyguaiacol (PDB entry 6ch6) (McGuire *et al.*, 2018 ▸), while the 5BR complex was consistent with a computationally hypothesized binding site (Barrios *et al.*, 2014 ▸), with both results together demonstrating that SFX provides accurate substrate-binding orientations.

**Figure 1:**
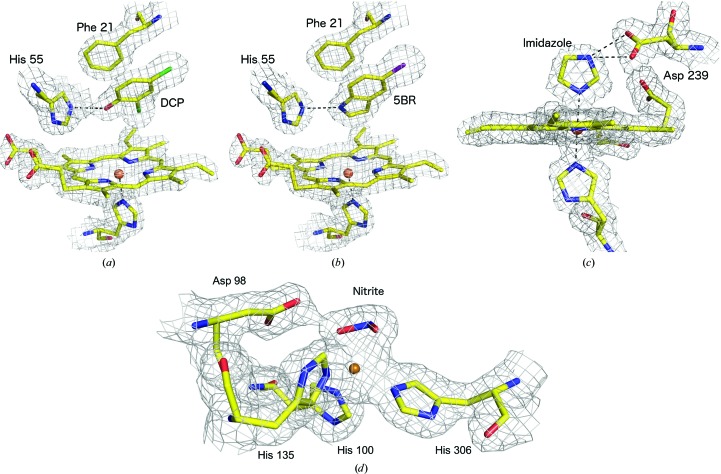
2*F*_o_ − *F*_c_ electron-density maps, contoured at 1σ, showing the complexes of DHP with (*a*) DCP with the Cl atoms shown in green and (*b*) 5BR with the Br atom shown in purple, (*c*) the complex of DtpAa with imidazole and (*d*) the complex of *Ac*NiR with nitrite. In each case, the active site of the monomer with the highest ligand occupancy is shown. The maps in (*a*)–(*d*) were generated using the all-image data sets.

The details of the binding modes themselves are beyond the scope of this manuscript and will be described in detail in a separate publication. Strong electron-density peaks were present for the two Cl atoms of DCP and the single Br atom of 5BR, allowing the ligand orientation to be easily confirmed, although it is important to note that the electron density was well defined for all atoms of the ligands. For both DHP–ligand structures one monomer had near-full occupancy, but significantly lower occupancy (as refined in *PHENIX*; Adams *et al.*, 2010 ▸) was observed in the second monomer of the homodimer [Figs. 1 ▸(*a*) and 1 ▸(*b*)]. This feature allowed us to examine the effect of ligand occupancy on ligand detectability in maps derived from SFX data (see below).

The SFX structure of DtpAa was determined in space group *P*2_1_ to 1.88 Å resolution (Table 1 ▸). The overall structure of the enzyme homodimer was highly similar to that of ferric DtpAa crystallized in a condition that did not contain imidazole (Ebrahim, Moreno-Chicano *et al.*, 2019 ▸). The examination of *F*_o_ − *F*_c_ difference maps indicated that an imidazole ligand was coordinated *via* an N atom to the distal position of the heme iron in one monomer of the DtpAa dimer with full occupancy. The Fe—N (imidazole) bond was 2.2 Å, while imidazole also formed two hydrogen bonds (2.7 and 2.9 Å) to Asp239 [Fig. 1 ▸(*c*)], and the heme pocket also contains several well ordered water molecules. Comparison with the ligand-free ferric DtpAa structure also obtained by SFX (Ebrahim, Moreno-Chicano *et al.*, 2019 ▸) revealed that the imidazole displaces the distal water molecule from the heme and induces a number of modest structural rearrangements in the heme pocket (Supplementary Fig. S2). A second imidazole molecule is bound to the protein away from the heme pocket, forming a 2.7 Å bond to Thr351 and interacting via a bridging water with Glu283. In contrast, for monomer *A* no imidazole ligand was observed in the distal heme pocket and instead a water molecule is bound at a distance of 2.4 Å in a similar manner to that in the ferric DtpAa structure (Ebrahim, Moreno-Chicano *et al.*, 2019 ▸).

The structure of *Ac*NiR in complex with nitrite was determined to 1.90 Å resolution (Table 1 ▸). The type 2 Cu site, which is the site of ligand binding, displayed clear electron density for a bound nitrite molecule with a bidentate O-binding geometry as previously described in multiple 100 K and room-temperature structures obtained from single crystals (Meyder *et al.*, 2017 ▸; Horrell *et al.*, 2016 ▸) [Fig. 1 ▸(*d*)]. The positions of the ligand-binding sites within the protein fold for each complex are shown in Supplementary Fig. S3.

### 3.3. Ligand density features as a function of the number of diffraction patterns in a data set   

**Figure 2:**
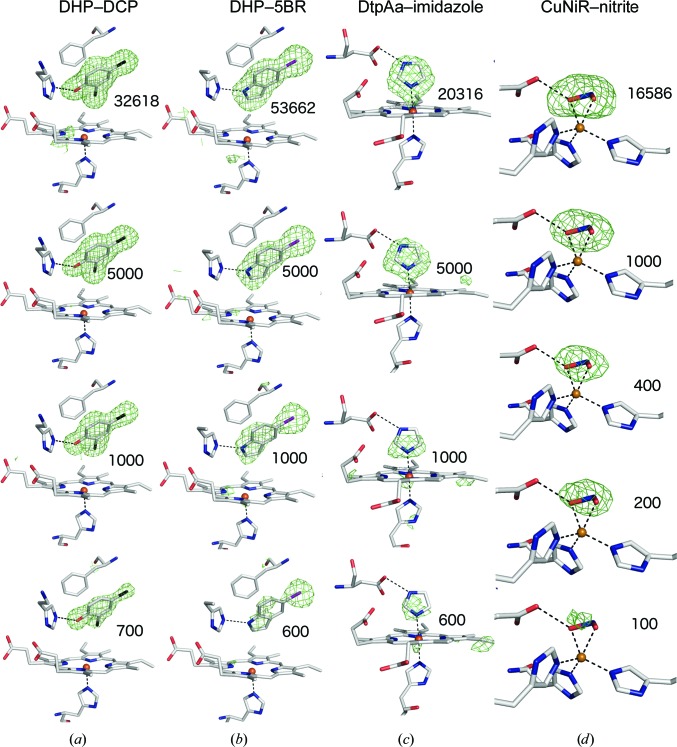
*F*_o_ − *F*_c_ simulated-annealing OMIT maps contoured at 3σ for the heme region from selected data subsets for (*a*) DHP–DCP, (*b*) DHP–5BR, (*c*) DtpAa–imidazole and (*d*) *Ac*NiR–nitrite, each superposed on the refined structure from all data. For (*a*) and (*b*) the highest occupancy ligand monomer of the homodimer is shown. Additional subsets are shown in Supplementary Figs. S5, S6 and S8–S11.

As described above, all three ligands were unambiguously identified in maps derived from the full data sets, demonstrating that ample diffraction patterns had been included in the merged data sets, which had good data-quality metrics (Table 1 ▸). To test the lower limits of the number of diffraction patterns that would allow us to identify bound ligands in high-throughput SFX experiments, the data were partitioned into subsets of decreasing size to produce independent merged data sets containing progressively fewer diffraction patterns, (Supplementary Tables S1–S4). OMIT difference maps were generated by simulated-annealing refinement in *PHENIX* (Adams *et al.*, 2010 ▸) using the all-data structure with ligand atoms omitted in the initial model. As expected, the merging and refinement statistics, and consequently the resolution cut-off, progressively deteriorated as the number of merged patterns was reduced (Fig. 2 ▸, Supplementary Tables S1–S4, Supplementary Fig. S4). The OMIT map quality was, as might be reasonably expected, proportional to the number of images in the data set. As a simple practical test to emulate a typical crystallographic workflow, the ‘Find Ligands’ feature of *Coot* (Debreczeni & Emsley, 2012 ▸; Emsley, 2017 ▸) was used to test whether each ligand could be correctly fitted into the simulated-annealing *F*_o_ − *F*_c_ map without bias from the experimentalist’s prior knowledge of the correct pose.

### 3.4. 2,4-Dichlorophenol–DHP complex   

We first examined the effect of the number of crystals included in a data set on the resulting electron-density maps for the complex between DHP and DCP. Very clear *F*_o_ − *F*_c_ simulated-annealing OMIT map features for the ligand were evident in subsets considerably smaller than the ‘full’ data sets. For example, a subset of 5000 crystals showed merging statistics that would still be considered acceptable by standard assessments [*R*_split_ = 0.17 (0.73), CC_1/2_ = 0.95 (0.56) to 1.95 Å resolution] and unsurprisingly ligand finding was straightforward. When the data set was reduced to containing only 1000 crystals the merging statistics were poor [*R*_split_ = 0.39 (0.65), CC_1/2_ = 0.72 (0.57) to 2.2 Å resolution], and with 500 images these metrics indicated very poor or even meaningless data quality [*R*_split_ = 0.56 (0.92), CC_1/2_ = 0.57 (0.42) to 2.2 Å resolution]. The refinement statistics also deteriorated with decreasing data-set size (Supplementary Table S1).

**Figure 3:**
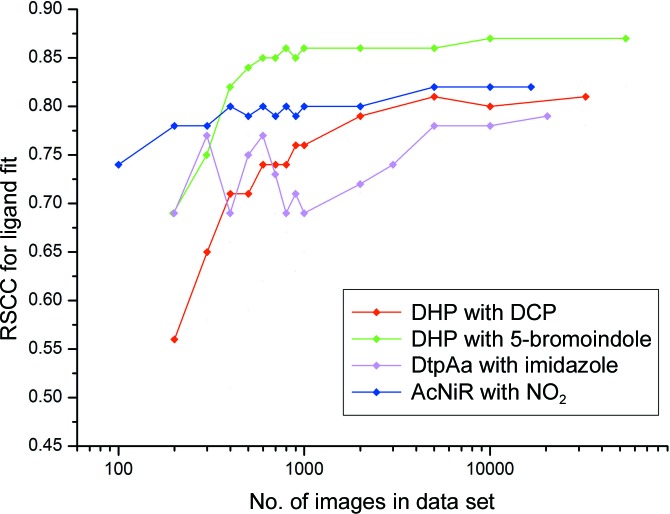
Real-space correlation coefficient (RSCC) values from *EDIAscorer* (Meyder *et al.*, 2017 ▸) as a function of the number of images per subset. Data are shown for the highest occupancy binding site for each complex. A plot including values for additional binding sites is shown in Supplementary Fig. S7.

Remarkably, data sets com­prising far fewer than 1000 indexed patterns displayed very clear features in simulated-annealing OMIT maps of the distal pocket, covering all atoms of the best-ordered DCP ligand (in monomer *B*). Examples are shown in Supplementary Fig. S5, where the *F*_o_ − *F*_c_ OMIT map allowed all atoms of the ligand to be unambiguously modelled, even when the merging statistics were very poor and refinement *R* factors were high (Supplementary Table S1). Because of the poor merging statistics with <1000 images, it was not possible to use these metrics to assess the resolution limit in merging for these data; however, refinement using the same resolution limit as the 1000-image set still allowed straightforward ligand placement. For data sets produced from <400 crystals, difference map quality rapidly deteriorated (Supplementary Fig. S5). This deterioration of the maps appears to approximately coincide with a loss of data completeness and redundancy in these data sets. The lower occupancy ligand present in the second monomer failed to be located in data-subset OMIT maps of decreasing size more rapidly than was the case for the fully occupied ligand (Supplementary Fig. S6). *EDIAscorer* (Meyder *et al.*, 2017 ▸) electron-density analysis is shown in Fig. 3 ▸ and Supplementary Fig. S7, showing the excellent quality of the difference map for all ligand atoms down to very low image numbers.

### 3.5. 5-Bromoindole–DHP complex   

Data and map quality followed a similar pattern with reducing crystal numbers to that described above for DCP (Table 1 ▸, Fig. 2 ▸, Supplementary Figs. S8 and S9). In this case, the lower occupancy of the two 5BR ligands was automatically found in *Coot* with a data set from 2000 images, but this step failed with 1000 images. For the higher occupancy 5BR ligand, the correct pose was found down to a data set of 800 images, while in data sets comprising 500, 600 or 700 images an incorrect pose was found by *Coot*, although manual re­orientation was straightforward based on the *F*_o_ − *F*_c_ map. A remarkable observation is that even in a data set comprising only 200 images (with 75.3% data completeness) the heavier Br atom of the ligand was clearly identified, with OMIT map peaks of 6.8σ (1.83 e^−^ Å^−3^) in monomer *B* and 4.3σ (1.16 e^−^ Å^−3^) in monomer *A* at its position (Supplementary Figs. S8 and S9).

### 3.6. Imidazole complex of DtpAa   

The imidazole ligand provided a more challenging case owing to its smaller size in comparison to DCP and 5BR and because of the lower symmetry space group (*P*2_1_) of the DtpAa crystals. For the latter reason, the merging statistics deteriorated more rapidly than for DHP (Supplementary Table S3). In particular, data completeness began to deterior­ate, with the 2000-image data set being essentially complete, while this was not the case for the 1000-crystal data set. With subsets of 5000 images or larger, *Coot* was able to successfully locate both the heme-coordinated imidazole and the second imidazole ligand located in the inter-monomer cleft [Supplementary Fig. S3(*c*)]. With smaller subsets, the latter ligand was not found, although the heme-bound imidazole was located in data sets of as few as 800 images. In these very small data sets the imidazole ring as positioned by *Coot* was sometimes rotated around its normal axis while still fitting the symmetrical electron-density feature well, but this was readily corrected by applying simple chemical knowledge, *i.e.* that N atoms rather than C atoms should be forming the coordination bond to the Fe atom and be oriented towards the Asp residue in the heme pocket. Simulated-annealing OMIT maps for the DtpAa–imidazole complex are shown in Fig. 2 ▸ and Supplementary Fig. S10, with electron-density statistics in Fig. 3 ▸ and Supplementary Fig. S7. We note that for all three ligands, even when automated ligand finding failed, significant ligand density was present that could allow manual identification in cases where the binding pocket was known in advance.

### 3.7. *Ac*NiR complex with nitrite   

Although nitrite is the smallest ligand of interest used in this study, *Ac*NiR has the inherent characteristic of crystallizing in a high-symmetry space group (*P*2_1_3), resulting in fewer data being required for a complete data set owing to the high redundancy of the data collected (Table 1 ▸). Again, very clear *F*_o_ − *F*_c_ simulated-annealing OMIT map features for the ligand were evident in subsets of small numbers of diffraction patterns, despite exhibiting merging statistics that would typically be considered rather poor (Figs. 2 ▸ and 3 ▸, Supplementary Table S4 and Supplementary Fig. S11). *Coot* successfully located nitrite binding at the type 2 Cu active site in subsets of very few crystals, with 200 being the lowest number of crystals that were needed to successfully auto-find the nitrite ligand. Although *Coot* was unsuccessful at determining the ligand in the lowest crystal subset of only 100 crystals, positive electron density is still identifiable at the site where ligand binding is expected, although this did not allow for reasonable modelling of a ligand.

### 3.8. OMIT maps from simulated-annealing refinement against ligand-free structures of the native enzymes   

**Figure 4:**
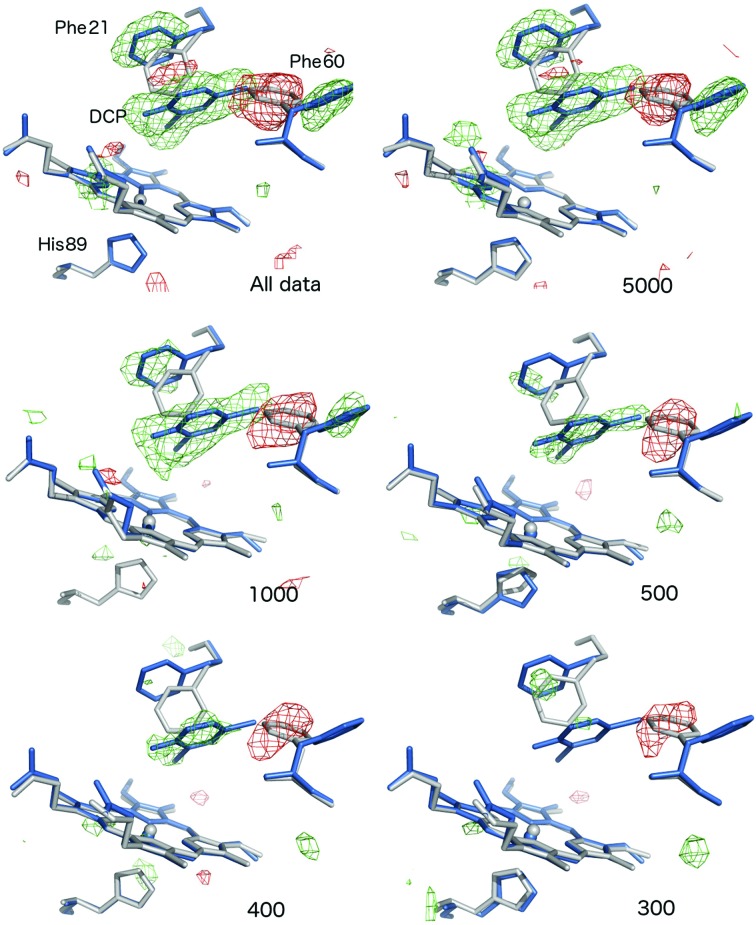
Difference map features produced by simulated-annealing refinement against ligand-free native structures clearly reveal ligand binding and active-site rearrangements in the absence of the risk of model bias. *F*_o_ − *F*_c_ OMIT maps, contoured at 3σ, are shown for DCP data subsets refined versus the native DHP structure. In each case, the native DHP structure from OMIT refinement versus a particular subset is shown in grey, while the superimposed structure of the ligand complex is shown in blue. Positive difference map features are shown in green, with negative features in red. Note that the flips of Phe21 and Phe60 to accommodate ligand binding, together with the ligand density itself, are very clearly defined in the data set obtained from all data and this is maintained in the 5000-image subset. Clear OMIT map features are apparent for Phe60 and DCP in data sets with as few as 400 images, while this was no longer the case in the 300-image subset.

Although simulated-annealing refinement as described above would reasonably be expected to remove all model bias, as an additional validation step selected subsets were refined against the corresponding native structures obtained by SFX (Ebrahim, Moreno-Chicano *et al.*, 2019 ▸; Moreno-Chicano *et al.*, manuscript in preparation), where the ligands were not present. OMIT map generation followed an identical procedure to that described above, with the exception of the input coordinate file used. The resulting OMIT maps are shown in Fig. 4 ▸ and Supplementary Figs. S12, S13 and S14) for data subsets of differing sizes. The results of this process corresponded well with the previously described OMIT maps, suggesting that model bias was not significant in the previous procedure for any of the complexes. Notably, for the two DHP ligand structures, in addition to very clear OMIT map density for the ligands themselves the map features clearly define the movements of heme-pocket residues that are necessary to accommodate the ligand (Fig. 4 ▸ and Supplementary Fig. S12). This provides further evidence of the information content within these data sets, despite the low numbers of diffraction patterns and extremely poor statistics. Importantly, we used *Ac*NiR–nitrite as a very challenging case to test the limitations of our approach as the nitrite ligand contains only three atoms and also displaces a water molecule upon binding (Antonyuk *et al.*, 2005 ▸). In addition, the water density in the native structure is disordered, with the presence of a second water molecule a possibility. Notably, refinement of *Ac*NiR data and subsets versus the native *Ac*NiR SFX structure produced clear positive difference map features for the nitrite atoms that are separated from the water molecule present in the native structure (Supplementary Fig. S14). For comparison, SA OMIT maps produced from refinement of the same subsets against the native *Ac*NiR SFX model with the copper-coordinated water molecule deleted are shown in Supplementary Fig. S15.

### 3.9. Detection of differences between ligands from *F*_o_ − *F*_o_ isomorphous difference maps   

**Figure 5:**
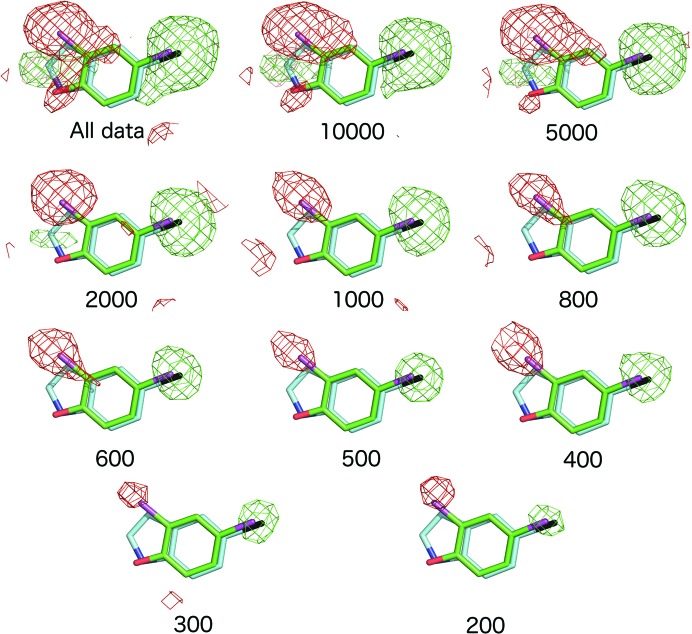
*F*_o_ − *F*_o_ isomorphous difference maps comparing the 5BR and DCP ligand complexes of DHP. Maps are *F*_o_(5BR) − *F*_o_(DCP) contoured at 3σ. With all data included, the map shows a clear positive peak near to the position of the Br atom of 5BR (black) and one of the Cl atoms of DCP (magenta), consistent with the greater number of electrons in bromine. A negative peak is present at the position of the second Cl atom of DCP, where the closest atom of 5BR is a carbon. An additional but weaker positive peak is present close to the C5 atoms of 5BR where no atom is present in DCP.

For the DHP case, in which two different ligands bind in a similar binding pose to the same enzyme pocket, we tested the ability to distinguish between these ligands using *F*_o_ − *F*_o_ isomorphous difference maps. For the full data sets, an *F*_o_(DHP–5BR) − *F*_o_(DHP–DCP) map is shown in Fig. 5 ▸. Strong positive density (a 32σ peak) is present where the Br atom of 5BR occupies a similar position to a Cl atom of DCP, consistent with the larger number of electrons on the Br atom. A negative feature is present over the second Cl atom of DCP, consistent with a C atom occupying a similar position in 5BR. Finally, a positive peak is present for the C5 atom of 5BR where no equivalent atom is present in DCP. As the number of crystals in a data subset decreases, the map features become less prominent, with the C5 feature disappearing in subsets of 1000 crystals or smaller. However, the features corresponding to the Br and Cl atoms are remarkably still evident, albeit much weaker, in subsets comprised of as few as 200 crystals (Fig. 5 ▸).

## 4. Discussion   

### 4.1. High-throughput determination of ligand-bound SFX structures using fixed targets   

Our results indicate that high-quality SFX crystal structures that allow unambiguous ligand identification may be achieved using our fixed-target approach. This can be achieved using a small quantity of enzyme sample with high throughput and rapid switchover between different proteins and ligands. Typical data-collection times for complexes were 30–60 min using all data measured, and using these data sets ligand modelling was clear and unambiguous. In comparison to previously published data for ligand-binding experiments (Naitow *et al.*, 2017 ▸; Bublitz *et al.*, 2015 ▸), the fixed-target approach allows the high-throughput production of multiple intact enzyme–ligand complex structures. In addition, the soaking and data-collection strategy can be easily adapted and optimized for a synchrotron beamline using the same sample-loading and mounting system.

Our results cover several different ligand-binding scenarios, such as coordinate-bond formation to a heme iron (imidazole) or copper (nitrite) and noncoordinate ligand binding in a pocket (DHP ligands), with the latter being highly relevant to the binding of ligands to pharmacologically important proteins such as cytochromes P450. In each structure, binding sites are present with different occupancies, allowing a further test of the ability of the method to locate high- or low-occupancy ligands.

The limits of our ability to identify ligand binding were tested using the small ligands nitrite (46 Da) and imidazole (68 Da). Both of these are much smaller than the fragments used in fragment-based drug design (FBDD), where 200 Da is a typical molecular weight (Price *et al.*, 2017 ▸). In the case of *Ac*NiR, a particular challenge was that nitrite displaces a water molecule on binding. In *Ac*NiR structures determined from single crystals, distinguishing between the electron-density features of active-site waters and nitrite is challenging and requires high-resolution data (Antonyuk *et al.*, 2005 ▸; Horrell *et al.*, 2018 ▸). Nonetheless, our method allowed the identification of these ligands in subsets comprising a small fraction of the full data sets. Our results are therefore strongly indicative that the ligands used in FBDD will be readily detected using our approach.

### 4.2. What is the minimal quantity of data required to identify ligand-binding modes?   

Analysis of simulated-annealing OMIT maps generated from data subsets containing only a subset of merged diffraction patterns clearly demonstrates that only a small fraction of the total data-collection time that we used is in fact necessary to locate ligands in the correct binding pose. For example, for the 5BR complex of DHP a subset of just 800 indexed images (∼1.5% of the total number of images in the full data set) was sufficient to correctly model the ligand using a careful strategy to preclude the possibility of model bias. A conservative approach of measuring several times this minimal number would still require only a small proportion of the 25 600 crystal positions on each chip. Our data also show that useful information is contained in data sets obtained from extremely small numbers of microcrystals; for example, the Br atom of 5BR was identified in a data set of only 200 crystals (<0.4% of the total data set).

In a lower symmetry space group (DtpAa; *P*2_1_), the ability to detect ligand binding in data sets of <2000 images was compromised by a lack of data completeness at higher resolution, although ligand finding was still achieved with 800 images. Notably, for the DHP structures in space group *P*2_1_2_1_2_1_ data completeness remained good in very small data sets; for example, for the DCP complex the 400-image data set retained >90% completeness in the highest resolution shell.

The high completeness of data sets formed from (relatively) small numbers of crystals parallels the success in forming complete data sets from multiple thin wedges in virus crystallography (Fry *et al.*, 1999 ▸). The completeness of the final data set is a function of the number of wedges collected and the point group of the crystals used, with the prerequisite for each approach being that the crystals must be randomly orientated. The completeness of the data obtained from small numbers of crystals here illustrates that this is the case for DtpAa, DHP and *Ac*NiR crystals on silicon chips. The bandwidth of the XFEL beam allows complete data to be obtained from fewer crystals than would be the case with a more monochromatic beam, yet still requires many more crystals than might be required in a wide-bandpass Laue experiment (Meents *et al.*, 2017 ▸). Our data strongly suggest that data completeness is the key metric for assessing the suitability of data sets for ligand-binding studies and that very poor values of other typically used metrics of data quality (for example CC_1/2_ and *R*_split_) still allow successful ligand characterization provided that the data are complete. For *Ac*NiR, with cubic symmetry, the data remained essentially complete in all of the subset sizes analysed, with density for the nitrite ligand remaining apparent down to <200 indexed images. We note that substantially more diffraction patterns would be required to obtain complete data on a monochromatic beamline.

More broadly, our data clearly show that substantial information content is present in noisy and apparently low-quality data sets derived from small numbers of merged diffraction patterns with very poor merging and refinement statistics. For example, a data set formed of 200 patterns revealed a very clear peak for the Br atom of the 5BR ligand (outer shell completeness 70.9% in DCP). Importantly, refinement of data subsets against native structures unambiguously showed not only clear density for ligands, but also any movements of the active-site residues needed to accommodate ligand binding (Fig. 4 ▸ and Supplementary Fig. S12). This provides conclusive evidence that the ligand density that we describe is not owing to model bias from prior knowledge of the binding mode.

### 4.3. Future potential of the ‘chip-soak’ approach for high-throughput structure determination of protein–ligand complexes   

In this work, SFX structures were recorded from two (*Ac*NiR), three (DHP–DCP) or four (DHP–5BR and DtpAa–imidazole) chips, aiming for 1–2 structures per hour. The number of chips used for a single structure was subsequently seen to err significantly on the side of caution, as in all cases sufficient data for unambiguous ligand identification were available from significantly less than half a chip. Crucially, careful data analysis demonstrated that data sets comprising of no more than a few hundred to a few thousand indexed images are sufficient to correctly model ligands into clear difference density features. Thus, without modification of the approach or changes to the experimental conditions, an approximately 4–5-fold increased throughput of multiple protein–ligand structures per hour could easily be realized. Rapid on-site data analysis should allow on-the-fly decision making as to whether sufficient data have been collected for a particular soak and if a ligand is indeed bound. A key advantage of the fixed-target sample-delivery method is that switching between samples of different protein–ligand soaks is no more time-consuming than continuing with a chip of the same sample. With typical loading rates of approximately 30%, multiple ligand soaks could be carried out on a single redesigned chip, again drastically increasing throughput. As a further example, for systems where approximately 1000 hits would be sufficient, at the latter hit rate some eight ligand complexes could be characterized on a single chip.

The sample quantity required for our approach (in the range of 1.35–6.0 mg protein per data set) is less than required in liquid-jet approaches, although higher than has been reported for high-viscosity (LCP) injection systems at XFEL (Weierstall *et al.*, 2014 ▸) and synchrotron (Weinert *et al.*, 2017 ▸) beamlines. An additional factor is ligand consumption. In our case, without optimization to minimize sample consumption, the typical ligand quantities used were in the range 4–40 µmol.

Our system of work is applicable at other current and future XFEL sources, such as PAL (60 Hz repetition rate), SwissFEL (100 Hz) and LCLS (120 Hz), as well as SACLA (30 Hz). However, XFEL sources with very high repetition rates or complex pulse patterns (for example EuXFEL and LCLS-II) may require a modified or different approach. We have demonstrated that at a source with a modest repetition rate sufficient data for multiple, unrelated, protein–ligand structures may be obtained within a couple of hours. Increasing this level of throughput to ∼5–20 structures per hour at higher repetition-rate sources, or collecting fewer images per complex (see above) as is practical, would allow, for example, >200 structures to be determined in a single 12 h shift, similar to dedicated synchrotron beamlines. Fixed targets are also well suited to time-resolved crystallography of, for example, protein–ligand complexes using laser pump–probe methods (Schulz *et al.*, 2018 ▸) and it is important to note that in time-resolved experiments significantly more data may be required as crystals may contain a mix of states.

Another key advantage is that the chip approach allows us to test soaking protocols at synchrotron beamlines under identical conditions to those used at the XFEL in order to ensure that soaking does not damage crystals and also that ligands are bound, albeit in a radiation-damaged structure. At such high rates of sample delivery, automation of chip loading and robotic sample exchange will of course become increasingly important. Our work demonstrates the feasibility of high-throughput room-temperature ligand screening by SFX using microcrystals and is highly applicable to drug-discovery efforts, including in fragment-based drug design. Our approach would be of particular importance in cases where only small weakly diffracting crystals are obtained or when the enzyme–ligand complexes are radiation-sensitive. We have demonstrated the ability to identify ligand binding by our high-throughput approach using ‘conventional’ approaches to both refinement and ligand finding. Further data-analysis improvements to the ability to identify in particular low-occupancy ligands in FBDD could be achieved using a multi-data-set approach, for example in *PanDDa*, with subtraction of the ligand-free ground state (Pearce, Krojer, Bradley *et al.*, 2017 ▸) and with refinement against a composite of the ligand-free and ligand-bound structures (Pearce, Krojer & von Delft, 2017 ▸).

In conclusion, we demonstrate (i) a method to rapidly measure SFX data sets from protein–ligand complexes and to rapidly switch between ligands during beamtime, (ii) that data sets comprised of hundreds to a few thousands of diffraction patterns can be sufficient for unambiguous ligand identification and (iii) that even ligands smaller than those used in fragment-based drug design may be located using our approach. These data demonstrate the feasibility of high-throughput structure determination of protein–ligand complexes at XFEL sources.

## Supplementary Material

PDB reference: dehaloperoxidase B, complex with 2,4-dichlorophenol, 6i7f

PDB reference: complex with 5-bromoindole, 6i6g

PDB reference: dye-type peroxidase Aa, complex with imidazole, 6i7c

PDB reference: copper nitrite reductase, complex with nitrite, 6qwg

Supplementary figures and tables. DOI: 10.1107/S2052252519011655/mf5035sup1.pdf

## Figures and Tables

**Table 1:** Data-collection, processing and refinement statistics for full SFX data sets for enzyme–ligand complexes Values in parentheses are for the outermost resolution shell.

Structure	DHP–DCP	DHP–5BR	DtpAa–imidazole	*Ac*NiR–nitrite
Data collection and processing				
Space group	*P*2_1_2_1_2_1_	*P*2_1_2_1_2_1_	*P*2_1_	*P*2_1_3
Unit-cell parameters (Å, °)	*a* = 60.9, *b* = 67.2, *c* = 68.7, α = β = γ = 90	*a* = 61.0, *b* = 67.3, *c* = 68.8, α = β = γ = 90	*a* = 72.5, *b* = 68.0, *c* = 73.5, α = γ = 90, β = 105.6	*a* = 97.6, *b* = 97.6, *c* = 97.6, α = β = γ = 90
Chips used	3	4	4	2
Images collected	76800	102800	102800	51200
Indexed images merged	32618	53662	20316	16586
Unique reflections	24749	24840	56220	24729
Resolution (Å)	37.7–1.85 (1.90–1.85)	45.6–1.85 (1.90–1.85)	70.8–1.88 (1.93–1.88)	43.7–1.90 (1.93–1.90)
Completeness (%)	100 (100)	100 (100)	100 (100)	100 (100)
Multiplicity	579 (340)	907.7 (524.0)	101.6 (64.2)	3281.4 (2299.1)
CC_1/2_	0.99 (0.66)	1.00 (0.65)	0.96 (0.60)	0.99 (0.63)
*R*_split_ (%)	6.6 (61.9)	5.5 (66.6)	15.8 (63.9)	9.73 (58.61)
Refinement				
Resolution range (Å)	34.4–1.85	45.6–1.85	35.3–1.88	43.7–1.90
*R*_work_ (%)	16.8	16.7	13.9	13.7
*R*_free_ (%)	19.9	18.9	17.6	17.2
R.m.s.d., bond lengths (Å)	0.010	0.005	0.010	0.006
R.m.s.d., bond angles (°)	1.23	0.96	0.87	0.90
Ramachandran most favoured (%)	98.2	98.9	98.5	98.8
PDB code	6i7f	6i6g	6i7c	6qwg
