# Supplementary material for: High-throughput structures of protein–ligand complexes at room temperature using serial femtosecond crystallography
Source: IUCrJ. 2019 Oct 10;6(Pt 6):1074–85. doi: 10.1107/S2052252519011655 (PMC6830213; doi:10.1107/S2052252519011655)
Supplement: Supplementary file 1 [file m-06-01074-sup1.pdf]

IUCrJ

Volume 6 (2019)

Supporting information for article:

High-throughput structures of protein–ligand complexes at room temperature using serial femtosecond crystallography

Tadeo Moreno-Chicano, Ali Ebrahim, Danny Axford, Martin V. Appleby, John H. Beale, Amanda K. Chaplin, Helen M. E. Duyvesteyn, Reza A. Ghiladi, Shigeki Owada, Darren A. Sherrell, Richard W. Strange, Hiroshi Sugimoto, Kensuke Tono, Jonathan A. R. Worrall, Robin L. Owen and Michael A. Hough

Supplementary Table S1 – Data collection and processing values for selected partitioned datasets of **DHP-2,4-DCP** in space group $P2_12_12_1$. Dataset resolutions were assessed using the $CC_{1/2}$ and R_{split} parameters apart from where indicated as *, where the statistics no longer allowed for a meaningful assessment and the resolution was maintained at that of the 1000 image subset. Refinement values refer to simulated-annealing omit map refinements with the DCP ligand deleted. Refinement values refer to simulated-annealing omit map refinements with the 2,4-DCP ligand deleted, hence the 'all data' refinement parameters are not identical to those in Table 1 where the ligand was included.

Structure	**All data**	**10000**	**5000**	**2000**	**1000**	**800**	**700**	**600**	**500**	**400**	**300**
Scaling & merging											
Images merged	32618	9997	4998	1999	999	797	698	599	499	398	299
Unique reflections	24749	22180	21232	17010	14878	14807	14775	14695	14593	14136	12895
Resolution (Å)	37.7-1.85	37.7-1.92	37.7-1.95	37.7-2.1	37.7-2.20	37.7-2.2*	37.7-2.2*	37.7-2.2*	37.7-2.2*	37.7-2.2*	37.7-2.2*
Outer shell (Å)	1.88-1.85	1.95-1.92	1.98-1.95	2.14-2.10	2.24-2.20	2.24-2.20	2.24-2.20	2.24-2.20	2.24-2.20	2.24-2.20	2.24-2.20
Completeness (%)	100 (100)	100 (100)	100 (100)	100 (99.9)	99.9 (99.9)	99.7 (99.7)	99.5 (98.9)	97.8 (98.1)	(96.5)	93.9 (90.4)	86.9 (80.9)
Multiplicity	579 (340)	156.9 (101.9)	78.5 (50.8)	32.78(21.9)	17.6 (12.0)	14.0 (9.6)	12.1 (8.4)	10.4 (7.2)	8.2 (5.6)	6.4 (4.5)	5.1(3.0)
$CC_{1/2}$	0.993 (0.66)	0.979 (0.653)	0.953 (0.56)	0.756 (0.726)	0.723 (0.568)	0.714 (0.398)	0.669 (0.346)	0.644 (0.322)	0.572 (0.415)	0.482 (0.350)	0.425(0.376)
R_{split}	0.066(0.619)	0.121(0.655)	0.172(0.726)	0.271(0.568)	0.394 (0.645)	0.445(0.726)	0.478(0.777)	0.509(0.840)	0.560 (0.918)	0.612 (0.860)	0.660(0.880)
Refinement											
Resolution (Å)	30.6-1.85	34.4-1.92	34.4-1.95	34.4-2.10	34.4-2.20	34.4-2.20	34.4-2.20	34.4-2.20	34.4-2.20	34.4-2.20	34.4-2.20
Outer shell (Å)	1.93-1.85	2.02-1.92	2.05-1.95	2.26-2.10	2.37-2.20	2.37-2.20	2.37-2.20	2.37-2.20	2.37-2.20	2.37-2.20	2.37-2.20
R_{work} (%)	17.2 (44.0)	17.1 (27.6)	17.5 (29.8)	18.2 (23.1)	22.6 (26.7)	23.3 (24.0)	23.5 (28.6)	24.8 (28.5)	26.4 (28.2)	27.4 (31.7)	29.8 (32.5)
R_{free} (%)	20.7 (49.5)	24.0 (33.3)	23.0 (40.2)	24.0 (31.2)	30.2 (37.0)	33.6 (34.4)	32.1 (43.0)	33.9 (42.8)	36.2 (40.2)	37.7 (42.9)	40.2 (48.6)
RMSD bond (Å)	0.012	0.012	0.013	0.013	0.013	0.015	0.013	0.015	0.017	0.017	0.015
RMSD angle (º)	1.25	1.30	1.29	1.36	1.34	1.45	1.38	1.52	1.61	1.61	1.58
Ramachandran most favoured (%)	98.2	97.4	97.4	96.7	96.3	97.8	97.8	99.0	97.4	95.2	94.0

Supplementary Table S2 – Data collection and processing values for selected subsets of the DHP **5-bromoindole** dataset in space group $P2_12_12_1$. Dataset resolutions were assessed using the CC and R_{split} parameters apart from where indicated as *, where the statistics no longer allowed for a meaningful assessment and the resolution was maintained at that of the 1000 image subset. Refinement values refer to simulated-annealing omit map refinements with the 5-Br ligand deleted, hence the 'all data' refinement parameters are not identical to those in Table 1 where the ligand was included.

**Structure**	**All data**	**10000**	**5000**	**2000**	**1000**	**800**	**700**	**600**	**500**	**400**	**300**	**200**
Scaling and merging												
Images merged	53662	9994	4997	1998	997	798	698	598	499	397	298	197
Unique reflections	24840	22987	20653	18386	14942	14931	14906	14862	14768	14531	13962	11933
Resolution (Å)	45.6-1.85	37.8-1.90	37.8-1.97	37.8-2.05	37.8-2.20	37.8-2.20*	37.8-2.20*	37.82.20*	37.8-2.22*	37.8-2.22*	37.8-2.20*	37.8-2.20*
Outer shell (Å)	1.90-1.85	1.95-1.90	2.02-1.97	2.10-2.05	2.26-2.20	2.26-2.20	2.26-2.20	2.26-2.20	2.26-2.20	2.26-2.20	2.26-2.20	2.26-2.20
Completeness (%)	100(100)	100(100)	100(100)	100(100)	100(100)	99.9(100)	99.6(99.7)	99.3(99.0)	98.5(98.0)	96.1(94.7)	90.8(87.8)	75.3(70.9)
Multiplicity	907.7 (524.0)	189.7(127.5)	95.3 (65.2)	38.5(26.8)	19.7 (13.9)	15.7(11.1)	13.4(9.4)	11.0(7.7)	9.3 (6.6)	7.5 (5.3)	5.8(4.3)	4.1(3.1)
$CC_{1/2}$	1.00 (0.65)	0.97(0.56)	0.95 (0.59)	0.87(0.58)	0.75 (0.60)	0.69(0.34)	0.64(0.32)	0.62(0.34)	0.59 (0.37)	0.51 (0.35)	0.44(0.41)	0.39(0.44)
R_{split} (%)	5.5 (66.6)	12.8(76.0)	17.2 (63.3)	27.9(62.1)	39.3 (62.9)	45.2(0.754)	48.8(78.3)	52.0(80.2)	0.541 (0.778)	62.0 (86.7)	69.5(87.7)	75.1(75.5)
Refinement												
Resolution range (Å)	45.6-1.85	34.4-1.90	34.4-1.97	34.4-2.05	34.4-2.20	34.4-2.20	34.4-2.20	34.4-2.20	34.4-2.20	34.4-2.20	34.4-2.20	37.8-2.20
Outer shell (Å)	1.92-1.85	1.99-1.90	2.07-1.97	2.16-2.05	2.37-2.20	2.37-2.20	2.37-2.20	2.37-2.20	2.37-2.20	2.37-2.20	2.37-2.20	2.42-2.20
R_{work} (%)	18.6	19.0(34.1)	18.7(24.9)	20.8(26.6)	23.0(27.0)	25.5(28.9)	26.2(30.9)	28.8(31.2)	29.6(34.1)	31.5(32.4)	34.1(33.6)	34.5(32.0)
R_{free} (%)	21.4	21.6(33.2)	23.5(30.1)	24.9(33.6)	26.8(30.8)	30.7(38.3)	31.8(33.6)	34.6(37.2)	35.9(40.4)	37.9(39.5)	43.5(44.2)	39.4(46.9)
RMSD bond (Å)	0.005	0.004	0.007	0.003	0.003	0.003	0.03	0.004	0.003	0.003	0.004	0.004
RMSD angle (º)	0.9	0.8	1.0	0.7	0.6	0.7	0.6	0.7	0.7	0.7	0.8	0.8
Ramachandran most favoured (%)	98.5	98.2	98.5	98.5	98.9	98.9	98.9	98.5	97.8	97.8	94.8	92.6

Supplementary Table S3 – Data collection and processing values for selected partitioned datasets of DtpAa-imidazole in space group $P2_1$. Dataset resolutions were assessed using the CC and R_{split} parameters apart from where indicated as *, where the statistics no longer allowed for a meaningful assessment and the resolution was maintained at that of the 1000 image subset. Refinement values refer to simulated-annealing omit map refinements with the imidazole ligand deleted, hence the 'all data' refinement parameters are not identical to those in Table 1 where the ligand was included.

Structure	All data	10000	5000	3000	2000	1000	900	800	700	600	400	200
Scaling and merging												
Images merged	20316	9994	4997	2998	1997	998	898	799	698	599	399	199
Unique reflections	56220	54449	50380	40390	40235	37986	37416	36192	34525	32131	24352	11447
Resolution (Å)	70.8-1.88	36.4-1.92	36.4-1.95	37.0-2.10	37.0-2.10*	37.0-2.10*	37.0-2.10*	37.0-2.10*	37.0-2.10*	37.0-2.10*	37.0-2.10*	37.0-2.10*
Outer shell (Å)	1.93-1.88	1.95-1.92	2.00-1.95	2.16-2.10	2.16-2.10	2.16-2.10	2.16-2.10	2.16-2.10	2.16-2.10	2.16-2.10	2.16-2.10	2.16-2.10
Completeness (%)	100(100)	100(100)	100(100)	99.9(99.9)	99.5(99.2)	93.6(84.2)	92.1(86.7)	89.0(81.3)	84.7(75.3)	78.6(67.8)	58.5(45.0)	26.4(17.7)
Multiplicity	101.6 (64.2)	50.5 (33.1)	26.6 (17.6)	16.7 (11.5)	11.0 (7.6)	5.9 (4.2)	5.6 (4.0)	5.1 (3.7)	4.6 (3.4)	4.2 (3.2)	3.4 (2.8)	2.6 (2.3)
$CC_{1/2}$	0.96 (0.60)	0.92 (0.47)	0.83 (0.44)	0.72 (0.46)	0.61 (0.37)	0.42 (0.31)	0.40(0.321)	0.36(0.39)	0.32(0.40)	0.30(0.44)	0.20 (0.56)	0.07(0.23)
R_{split} (%)	15.8(63.9)	22.8(72.1)	32.5(70.9)	41.6(61.9)	51.3(72.0)	66.6(84.2)	69.0(88.1)	72.5(86.8)	85.1(99.1)	79.7(89.1)	89.7(102.4)	96.9(83.3)
Refinement												
Resolution range (Å)	35.3-1.88	35.9-1.92	35.9-1.95	35.7-2.10	35.7-2.10	35.7-2.10	35.7-2.10	35.7-2.10	35.7-2.10	35.7-2.10	35.4-2.10	37.0-2.10
Outer shell (Å)	1.91-1.88	2.01-1.92	2.04-1.95	2.21-2.10	2.21-2.10	2.18-2.10	2.18-2.10	2.18-2.10	2.18-2.10	2.18-2.10	2.20-2.10	2.31-2.10
R_{work} (%)	16.2(28.7)	15.7(22.0)	17.7(29.1)	21.7(23.3)	22.3(26.6)	27.5(30.9)	28.5(33.8)	29.4(33.2)	30.1(33.5)	31.5(36.6)	33.0(32.9)	30.7(34.0)
R_{free} (%)	20.7(29.8)	20.0(26.9)	21.6(37.4)	27.0(29.3)	25.9(30.1)	33.4(34.4)	35.2(45.0)	36.4(39.3)	36.2 (36.5)	39.9(51.7)	45.4(47.8)	49.5(59.1)
RMSD bond (Å)	0.014	0.016	0.011	0.006	0.003	0.003	0.004	0.003	0.004	0.004	0.005	0.009
RMSD angle (º)	1.4	1.4	1.1	1.0	0.6	0.6	0.6	0.6	0.6	0.7	0.8	1.2
Ramachandran favoured (%)	96.3	98.3	98.5	95.6	98.2	98.1	97.7	97.2	97.1	95.1	89.9	72.9

Supplementary Table S4 – Data collection and processing values for partitioned datasets of *Ac*NiR-nitrite in space group $P2_13$. Dataset resolutions were assessed using the CC and R_{split} parameters apart from where indicated as *, where the statistics no longer allowed for a meaningful assessment and the resolution was maintained at that of the 1000 image subset. Refinement values refer to simulated-annealing omit map refinements with the nitrite ligand deleted, hence the 'all data' refinement parameters are not identical to those in Table 1 where the ligand was included.

**Structure**	**All data**	**10000**	**5000**	**2000**	**1000**	**800**	**600**	**500**	**400**	**300**	**200**	**100**
Scaling and merging												
Images merged	16586	9988	4993	1997	996	795	597	497	398	298	199	98
Unique reflections	24729	24001	22575	20649	18162	18161	18160	18160	18160	18160	18159	18101
Resolution (Å)	43.7-1.90	43.7-1.92	43.7-1.96	43.7-2.02	43.7- 2.11	43.7- 2.11*	39.9- 2.11*	39.9- 2.11*	39.9- 2.11*	39.9- 2.11*	39.9-2.11*	39.9-2.11*
Outer shell (Å)	1.93-1.90	1.99–1.92	2.03–1.96	2.09–2.02	2.15 – 2.11	2.15 – 2.11	2.15 – 2.11	2.15 – 2.11	2.15 – 2.11	2.15 – 2.11	2.15 – 2.11	2.15 – 2.11
Completeness (%)	100 (100)	100 (100)	100 (100)	100 (100)	100 (100)	100 (100)	100 (100)	100 (100)	100 (100)	100 (100)	100 (100)	99.6 (99.6)
Multiplicity	3281.4 (2299.1)	2198.4 (1461.9)	1009.4 (713.3)	391.7 (276.0)	181.8 (128.7)	136.4 (96.6)	95.3 (67.3)	77.6 (54.8)	61.0 (43.0)	42.7 (30.1)	27.1 (19.2)	13.0 (9.3)
$CC_{1/2}$	0.99 (0.63)	0.98(0.62)	0.97(0.56)	0.92 (0.55)	0.92 (0.59)	0.84 (0.55)	0.82 (0.45)	0.81 (0.48)	0.77 (0.40)	0.71 (0.40)	0.61 (0.38)	0.4 (0.07)
R_{split} (%)	9.73 (0.59)	12.1 (62.5)	15.8 (61.8)	23.2 (70.1)	30.8 (68.9)	33.4 (73.5)	36.9 (87.7)	39.5 (91.0)	43.5 (105.1)	48.9 (110.0)	59.7 (129.5)	78.3 (160.1)
Refinement												
Resolution range (Å)	43.66-1.90	43.66-1.92	43.66-1.96	43.66-2.02	43.66-2.11	43.66-2.11	34.5-2.11	34.5-2.11	34.5-2.11	34.5-2.11	34.5-2.11	34.5-2.11
Outer shell (Å)	2.00-1.90	1.98-1.92	2.05-1.96	2.13-2.02	2.22-2.11	2.22-2.11	2.22-2.11	2.22-2.11	2.22-2.11	2.22-2.11	2.22-2.11	2.22-2.11
R_{work} (%)	13.9 (23.9)	14.7 (26.4)	15.4 (24.3)	16.3 (24.2)	17.8 (24.1)	18.4 (24.8)	19.2 (26.1)	19.9 (27.0)	20.7 (27.3)	22.4 (28.3)	24.3 (29.1)	28.6 (32.8)
R_{free} (%)	17.4 (32.0)	18.7 (32)	19.0 (28.2)	20.7 (25.9)	23.3 (28.1)	23.3 (27.4)	25.1 (29.8)	25.5 (31.5)	26.5 (33.4)	28.3 (32.8)	30.2 (33.8)	35.1 (40.0)
RMSD bond (Å)	0.007	0.009	0.004	0.005	0.005	0.004	0.004	0.004	0.005	0.004	0.006	0.006
RMSD angle (º)	0.90	0.93	0.76	0.79	0.81	0.77	0.77	0.80	0.80	0.76	0.81	0.84
Ramachandran favoured (%)	98.8	98.8	98.8	99.8	98.5	98.5	98.8	97.9	97.6	97.6	97.9	95.8

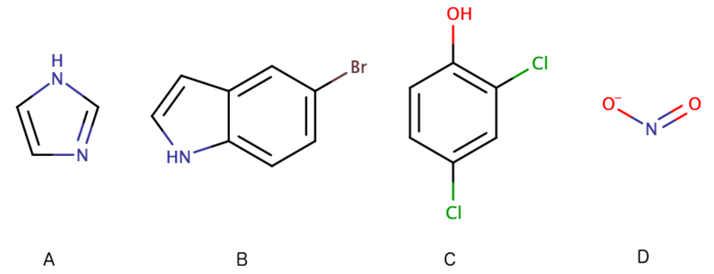

Supplementary Figure S1: Chemical structures of (a) imidazole, (b) 5-bromoindole, (c) 2,4-dichlorophenol and (d) nitrite. Structures were generated using MarvinSketch v19.6.0. Hydrogen atoms are omitted as these are not identified from the electron density maps. Molecular weights 68, 163, 195 and 46 Da, respectively.

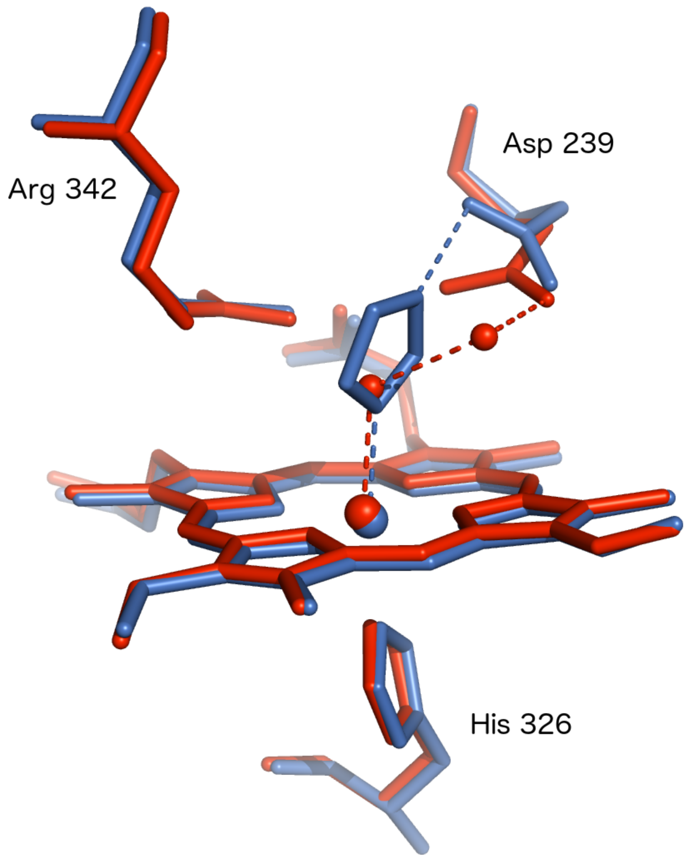

Supplementary Figure S2: Superposition of the two subunits of the DtpAa structure. In monomer B (blue) an imidazole ligand is bound to the heme Fe and forms a hydrogen bond to Asp 239. In monomer A (red) a water molecule is bound to the heme Fe with this water also forming bonds to a second distal pocket water and to Asp 239. We note that the monomer A structure is similar to that present in the ferric DtpAa structure (red, PDB 6I43).

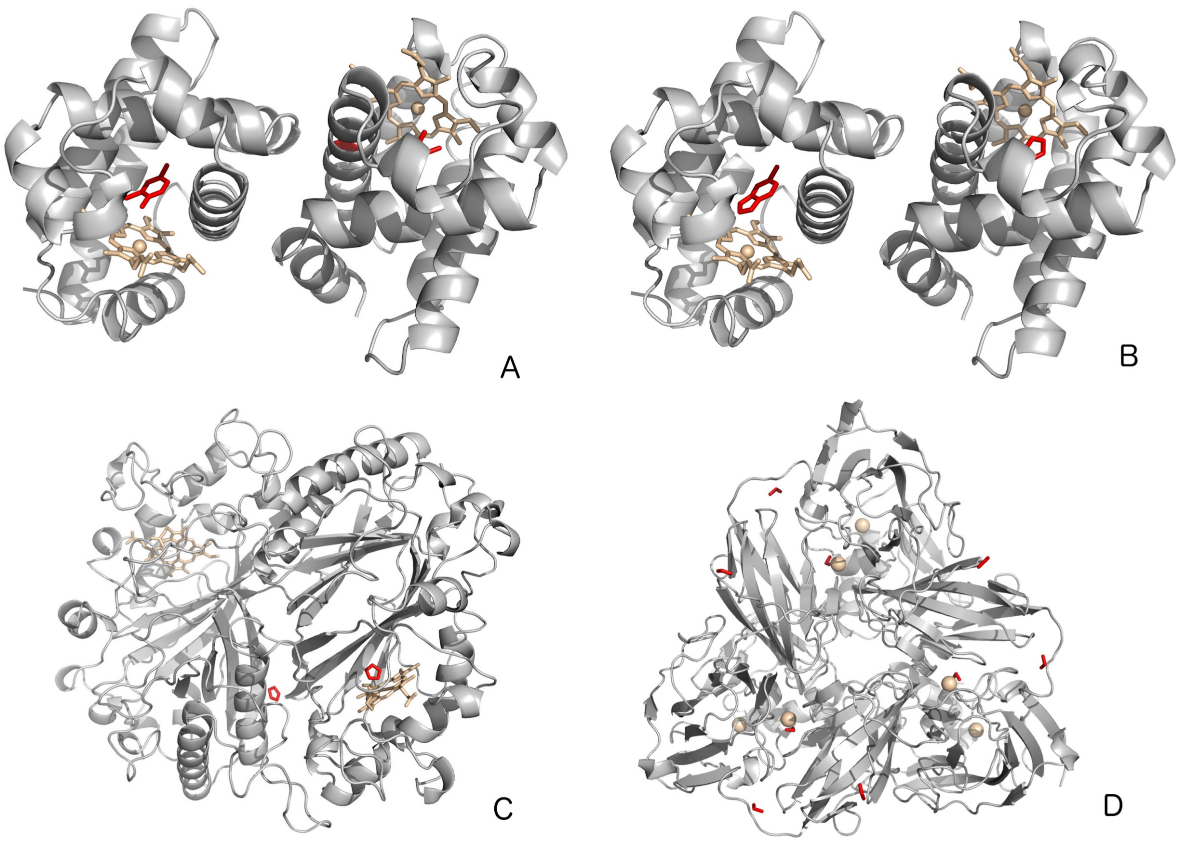

Supplementary Figure S3: Cartoon representation of protein structures with ligands shown in red and the metal centres in beige. (A) DHP-DCP, (B) DHP-5BR, (C) DtpAa-imidazole and (D) *Ac*NiR-nitrite. Note that in panel D the type 2 Cu site where nitrite binds is located at an inter-monomer interface so a symmetry generated trimer is shown.

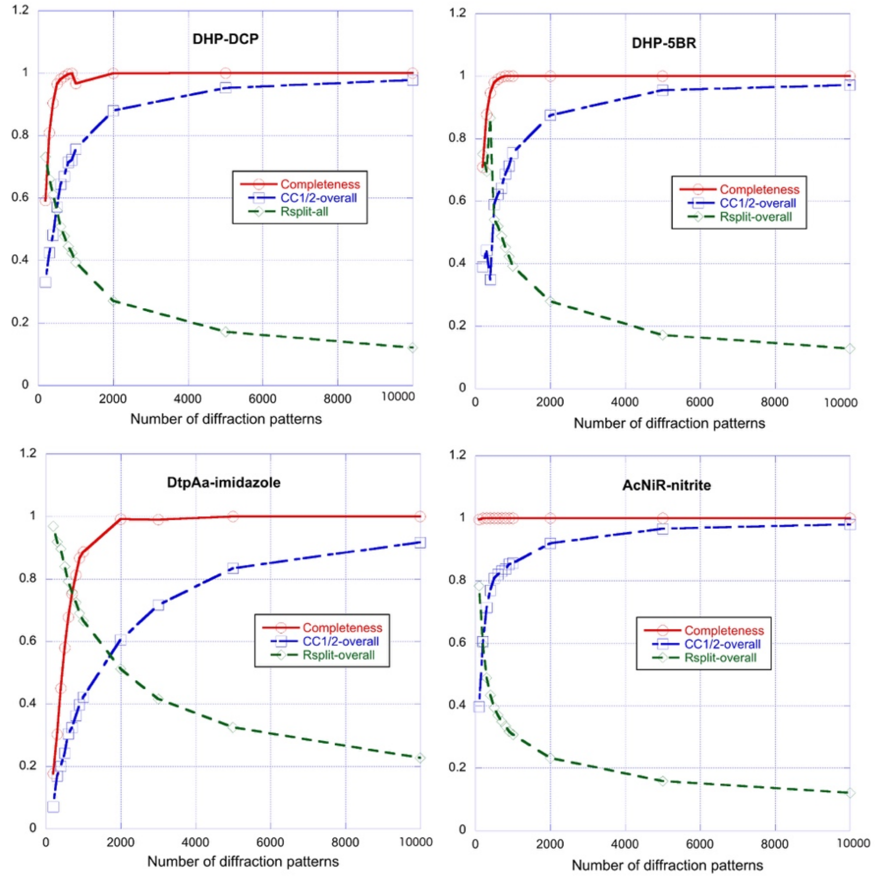

Supplementary Figure S4: Data quality metrics as a function of the number of merged crystals in data subsets. Lines connect data points and do not represent a function. Note that datasets were merged to differing resolutions as shown in Supplementary Tables 1-4. To facilitate comparisons, data are shown for datasets of 10000 crystals and smaller. Note that overall values for $CC_{1/2}$ and R_{split} are shown together with the completeness in the highest resolution shell.

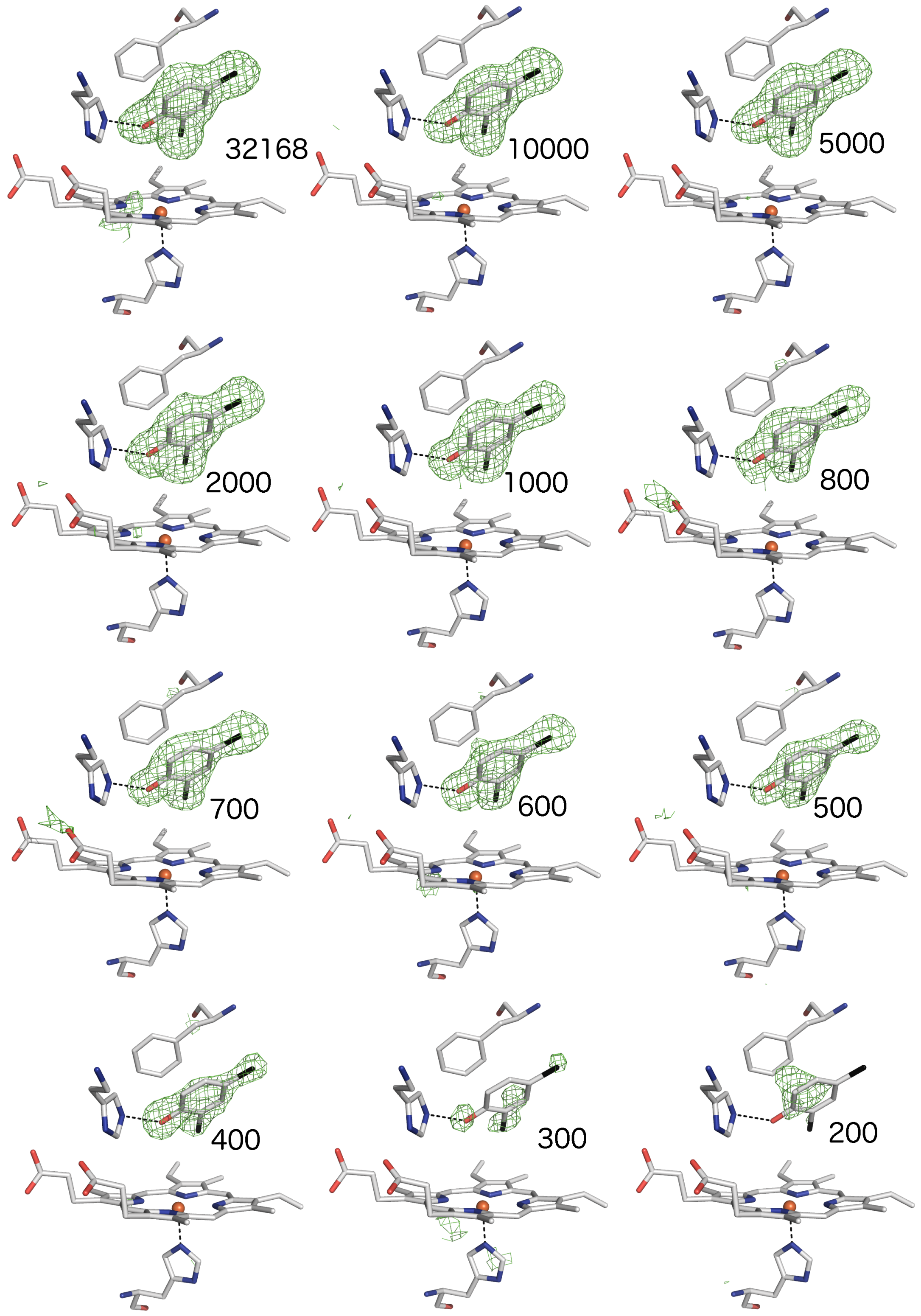

Supplementary Figure S5: *Fo-Fc* simulated annealing omit maps, contoured at 3σ from selected data subsets of the DHP-DCP structure. The heme pocket region in monomer B is shown, where the ligand occupancy was 0.95.

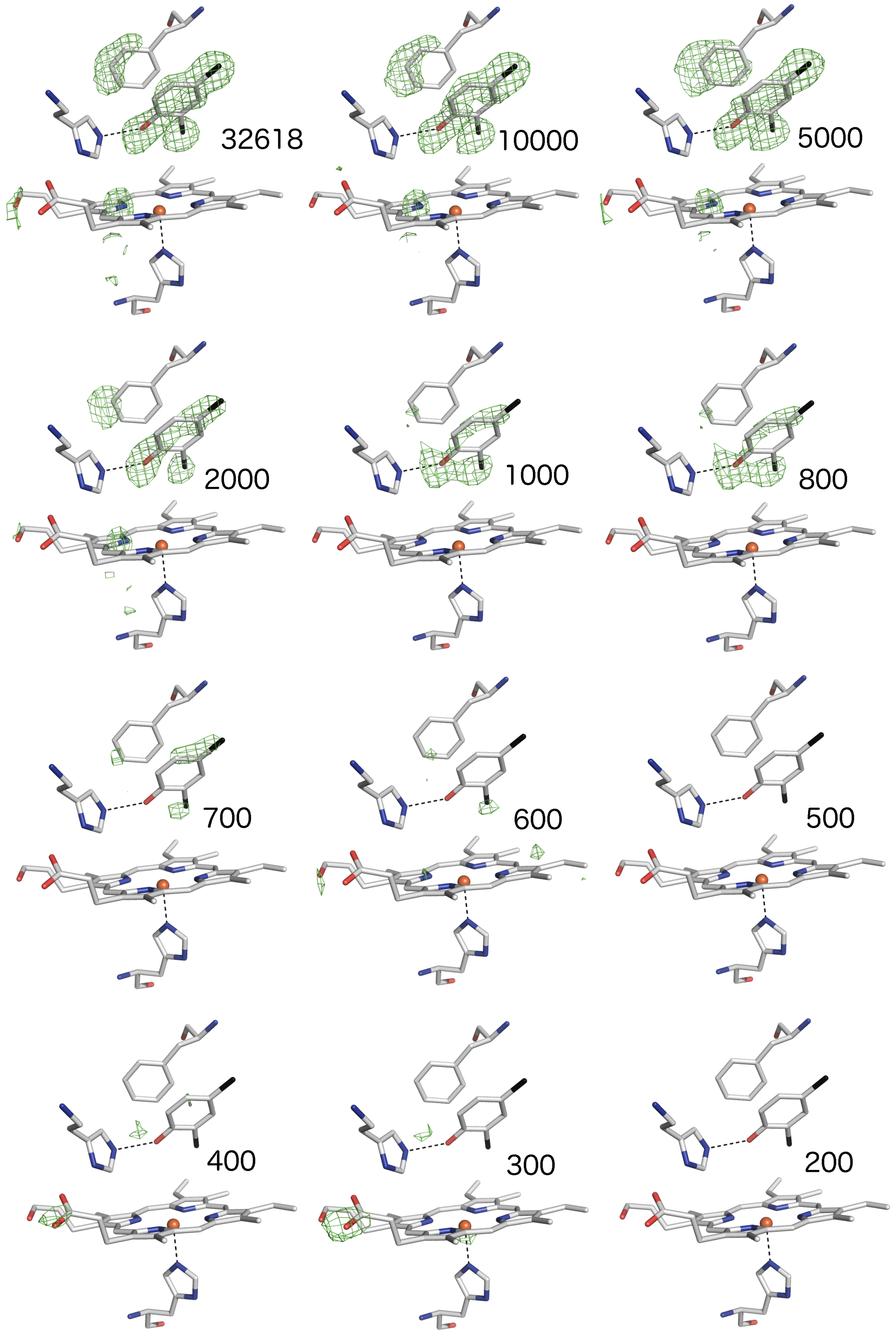

Supplementary Figure S6: *Fo-Fc* simulated annealing omit maps, contoured at 3σ from selected data subsets of the DHP-DCP structure. The heme pocket region in monomer A is shown, where the ligand occupancy was 0.59.

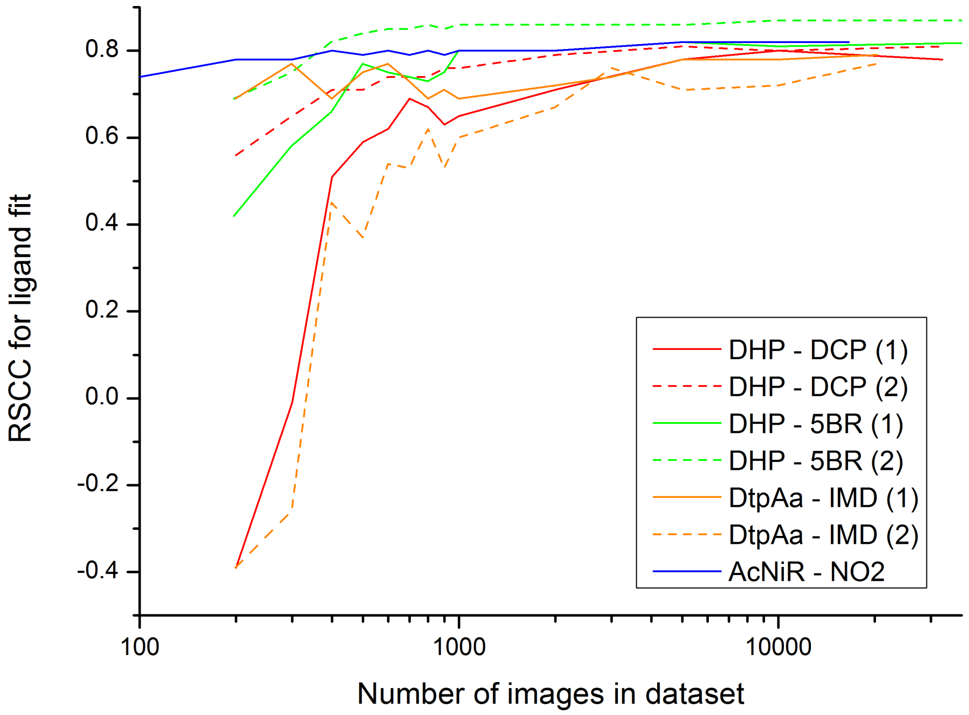

Supplementary figure S7: Real space correlation coefficient (RSCC) values for all ligand atoms as a function of the number of images per subset. Data are shown for the high and low occupancy binding sites for each complex. Values calculated using Ediascorer (Meyder et al. 2017).

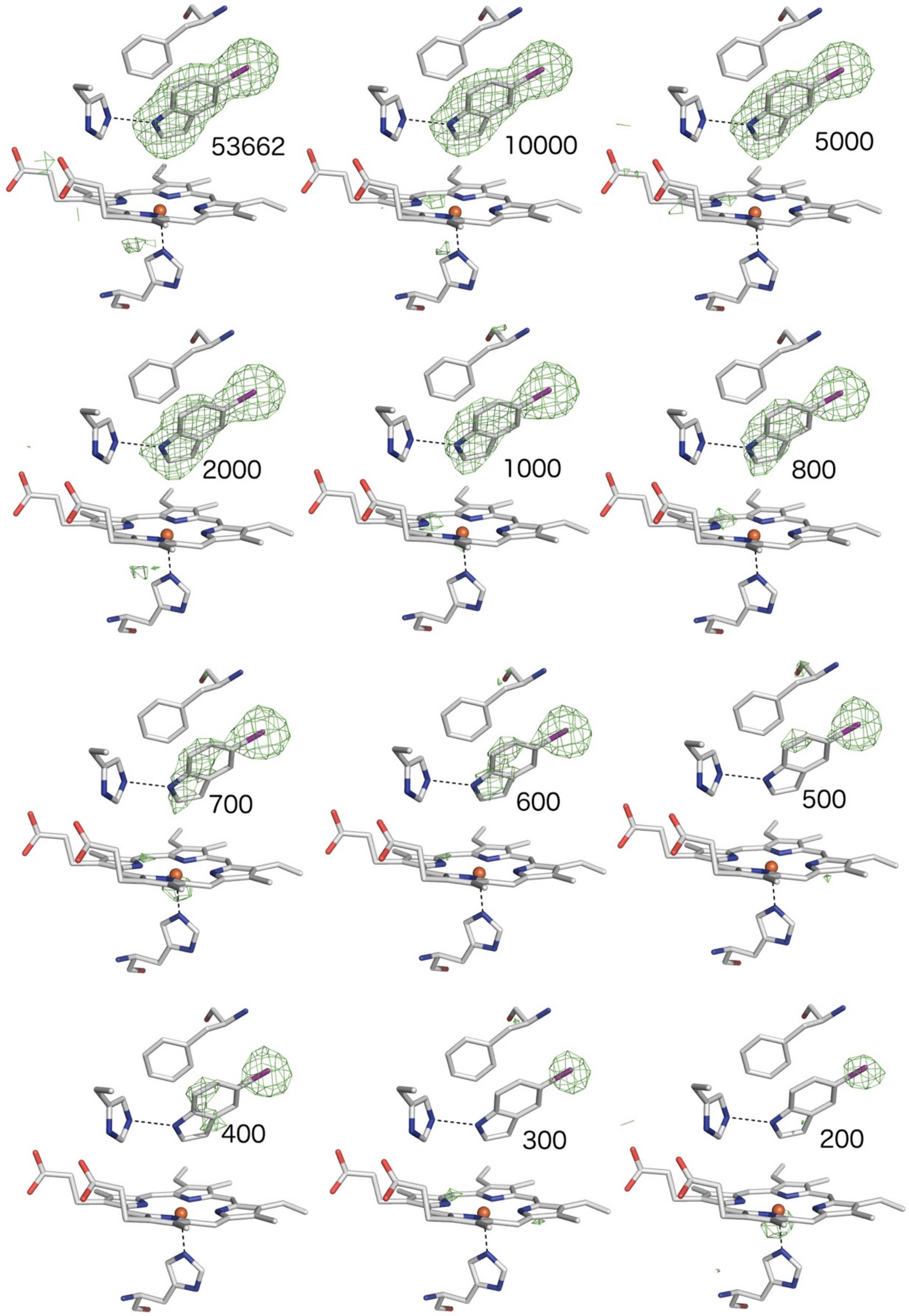

Supplementary Figure S8: *Fo-Fc* simulated annealing omit maps, contoured at 3σ from selected data subsets of the DHP-5BR structure. The heme pocket region in monomer B is shown, where the ligand occupancy was 0.96.

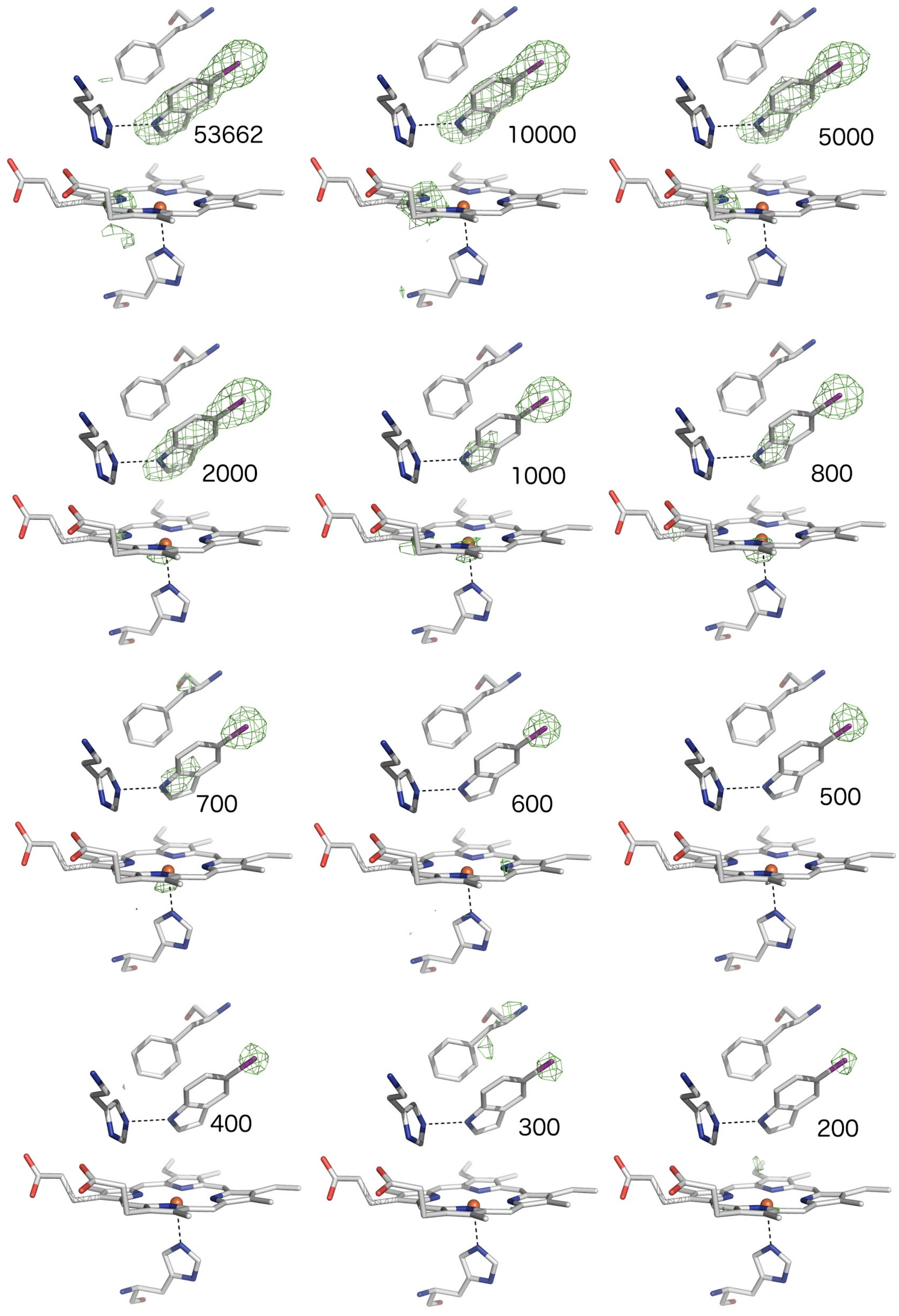

Supplementary Figure S9: *Fo-Fc* simulated annealing omit maps, contoured at 3σ from selected data subsets of the DHP-5BR structure. The heme pocket region in monomer A is shown, where the ligand occupancy was 0.6.

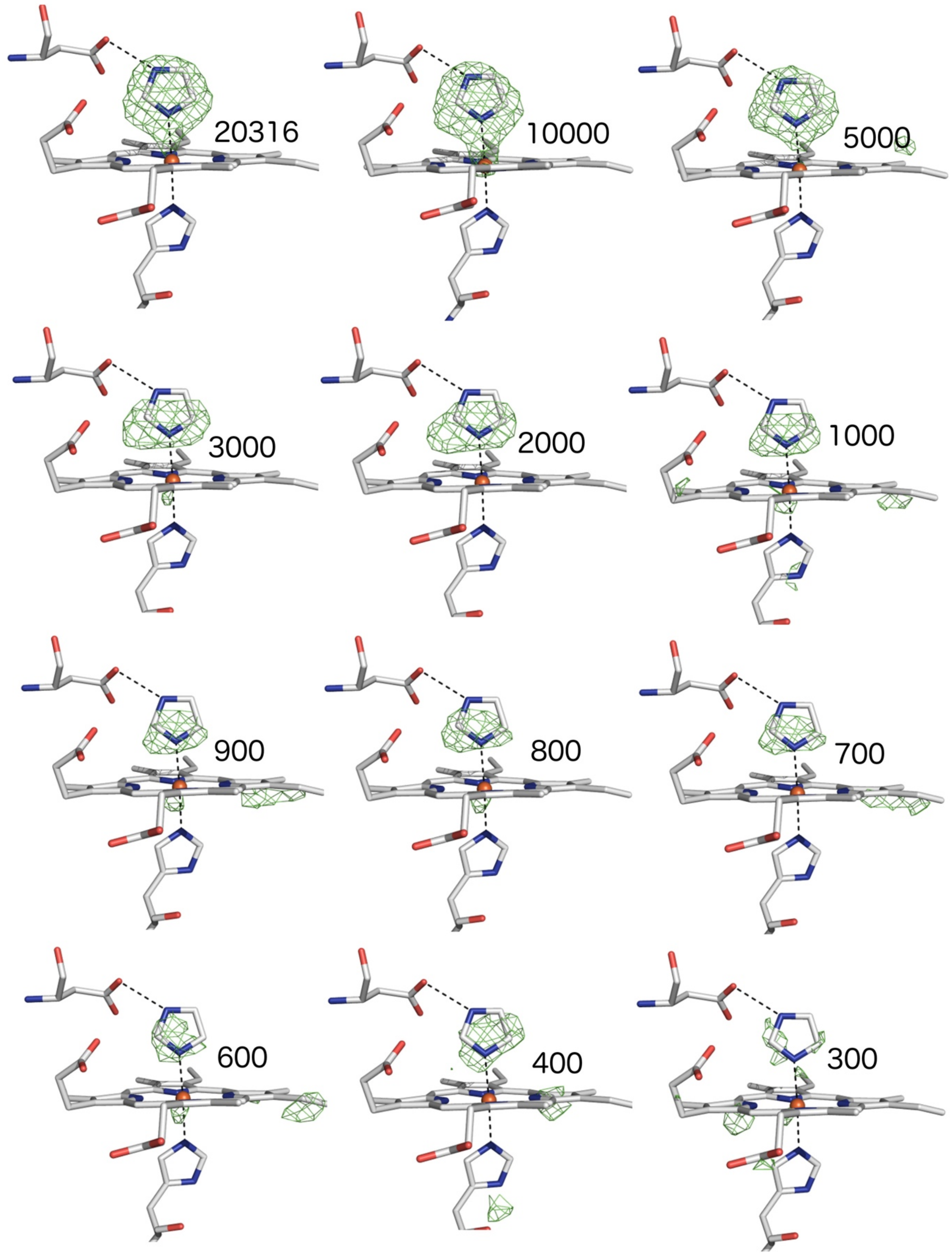

Supplementary Figure S10: *Fo-Fc* simulated annealing omit maps, contoured at 3σ from selected data subsets of the DtpAa-imidazole structure. The heme pocket region is shown, where the ligand occupancy was 0.98. The ligand bound structure was superimposed on the coordinate file from omit map generation using all heme atoms.

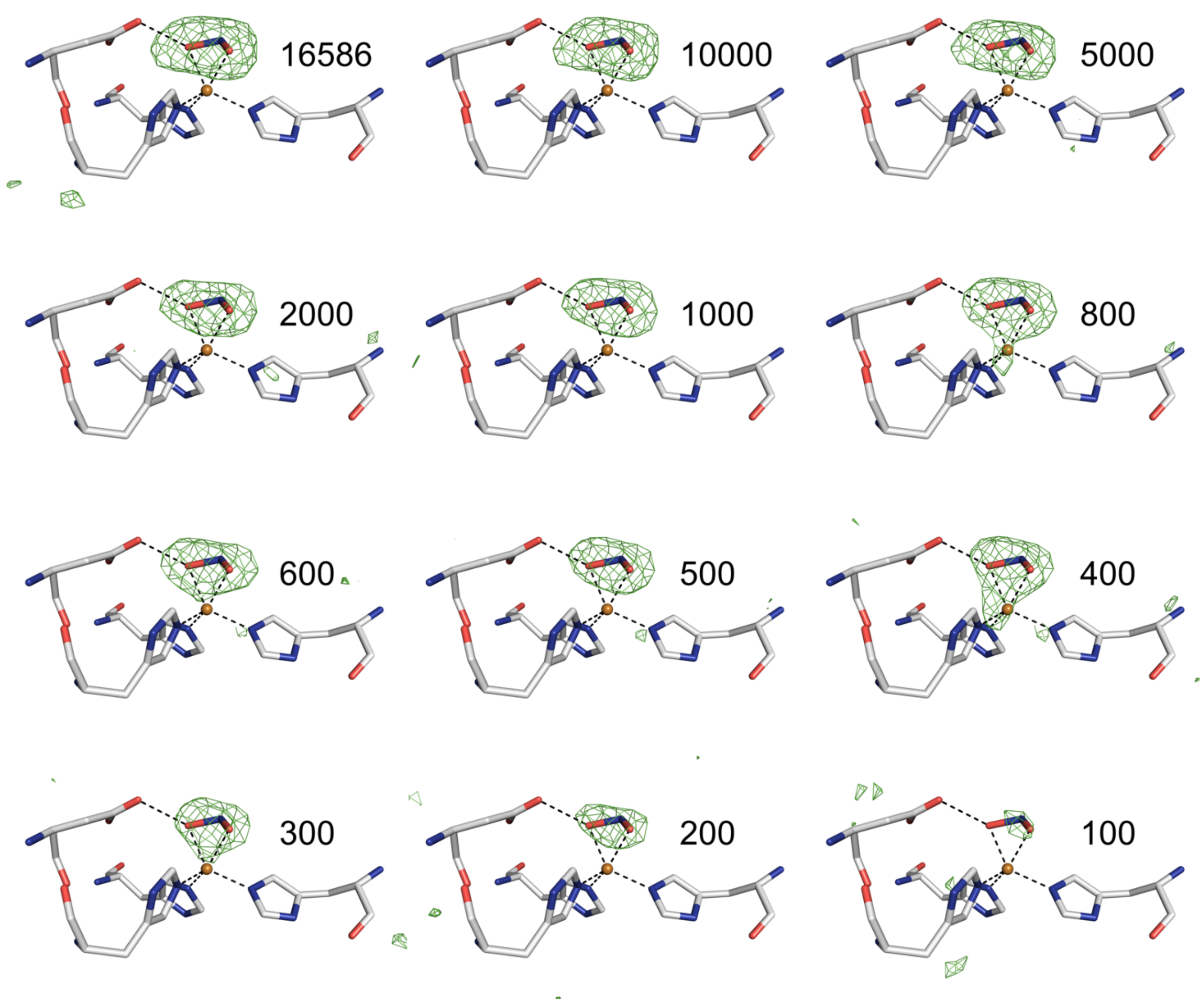

Supplementary Figure S11: *Fo-Fc* simulated annealing omit maps, contoured at 3σ from selected data subsets of the *Ac*NiR-nitrite structure. The type 2 Cu active site region is shown, where the nitrite ligand occupancy was 1.

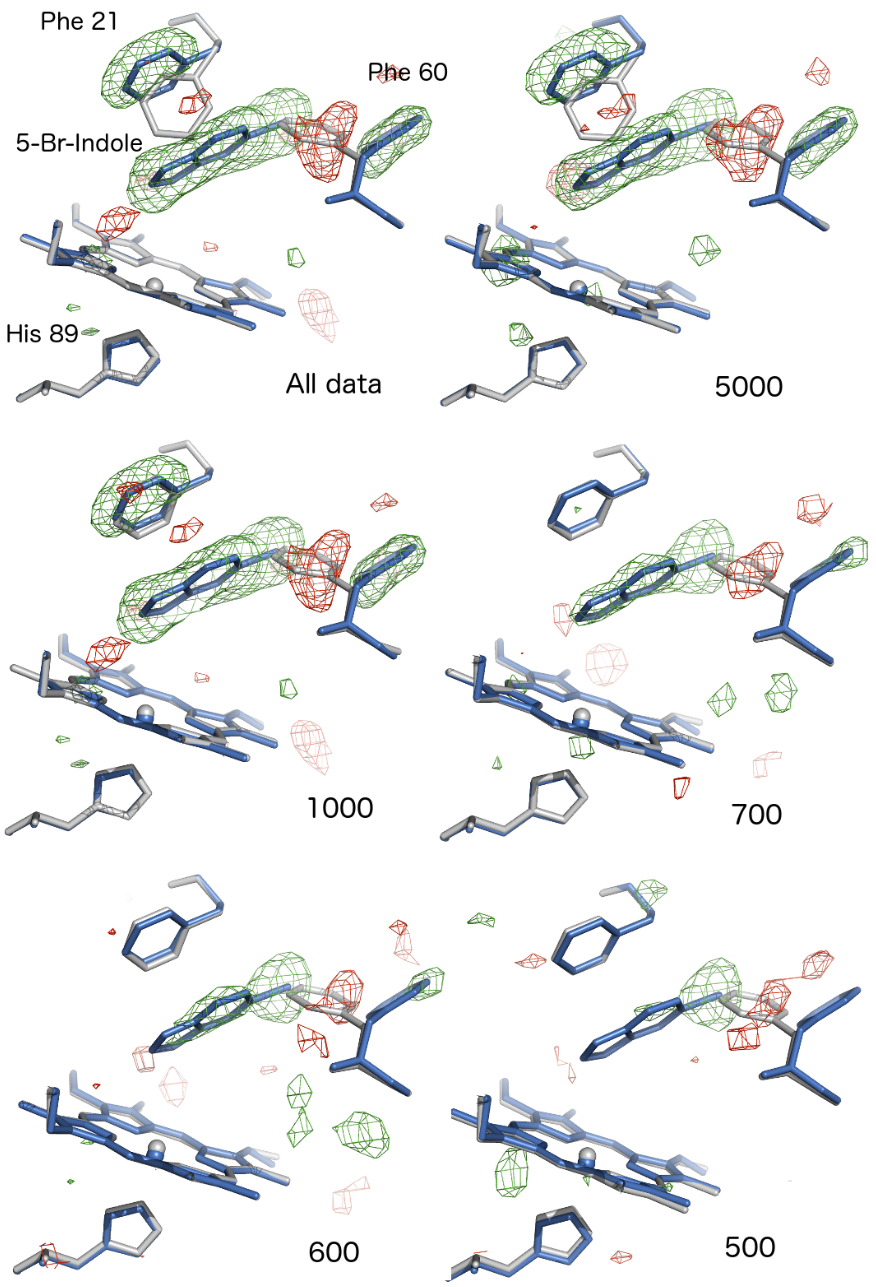

Supplementary Figure S12: 5BR dataset omit maps contoured at 3σ from simulated annealing refinement of DHP-5BR subsets versus the structure of native DHP. The ligand-bound structure for each case is coloured blue, the native DHP structure from omit refinement in grey. Note that the flip of residue Phe 60 to accommodate ligand binding, together with the ligand density itself are clearly defined in the dataset obtained from 2000 crystals. In the 5BR- 800 dataset the same omit map features remain present, albeit less clearly. In the 500 crystal dataset the position of the Br atom is apparent together with weak negative density for the incorrect Phe position.

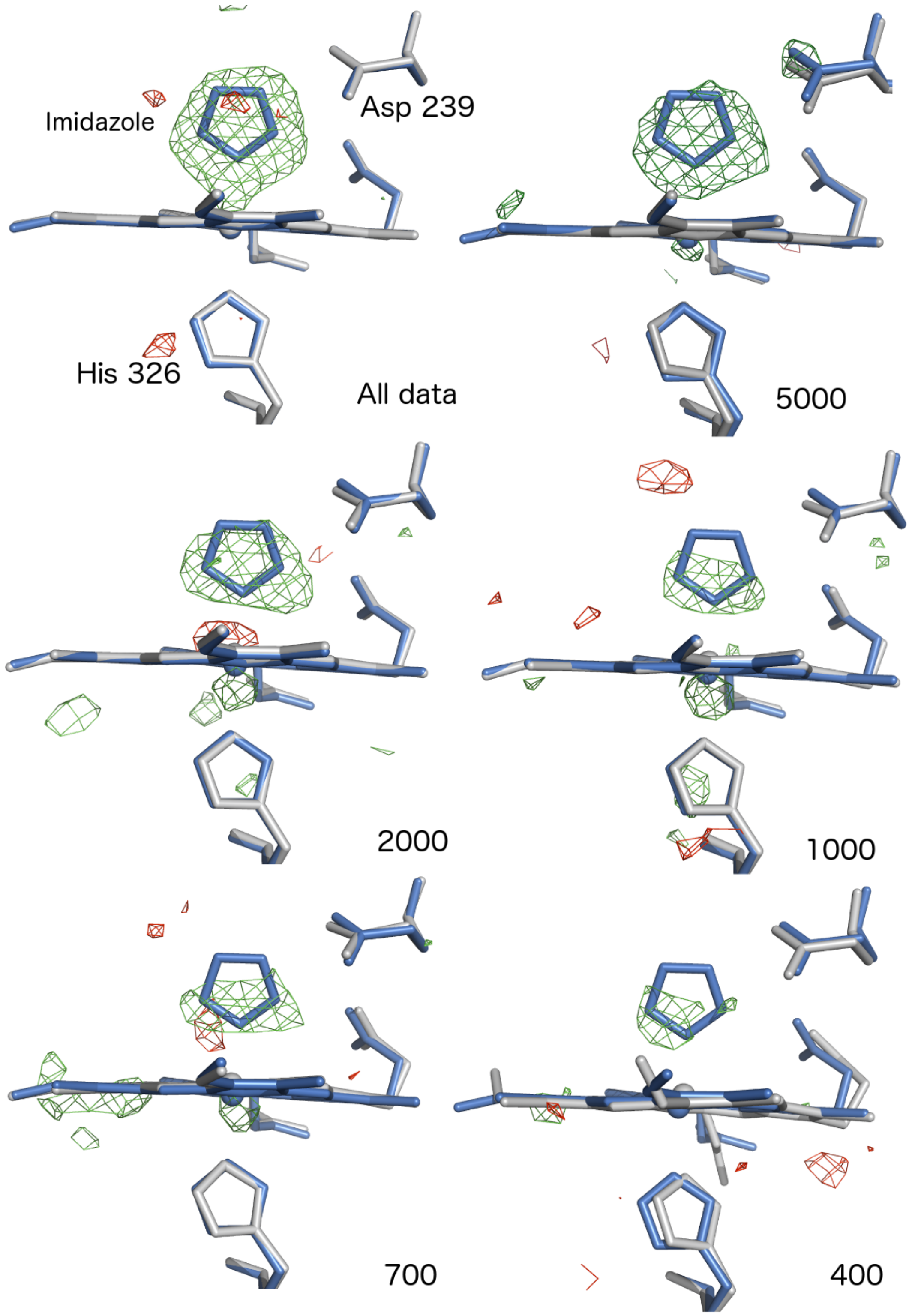

Supplementary Figure S13: *Fo-Fc* omit map, contoured at 3σ from simulated annealing refinement of DtpAa-imidazole subsets versus the structure of native DtpAa (Ebrahim *et al.* 2019). Clear positive density for the imidazole ligand is evident. The structure from omit refinement is shown in grey, with the superimposed all-data structure of the DtpAa imidazole complex shown in blue.

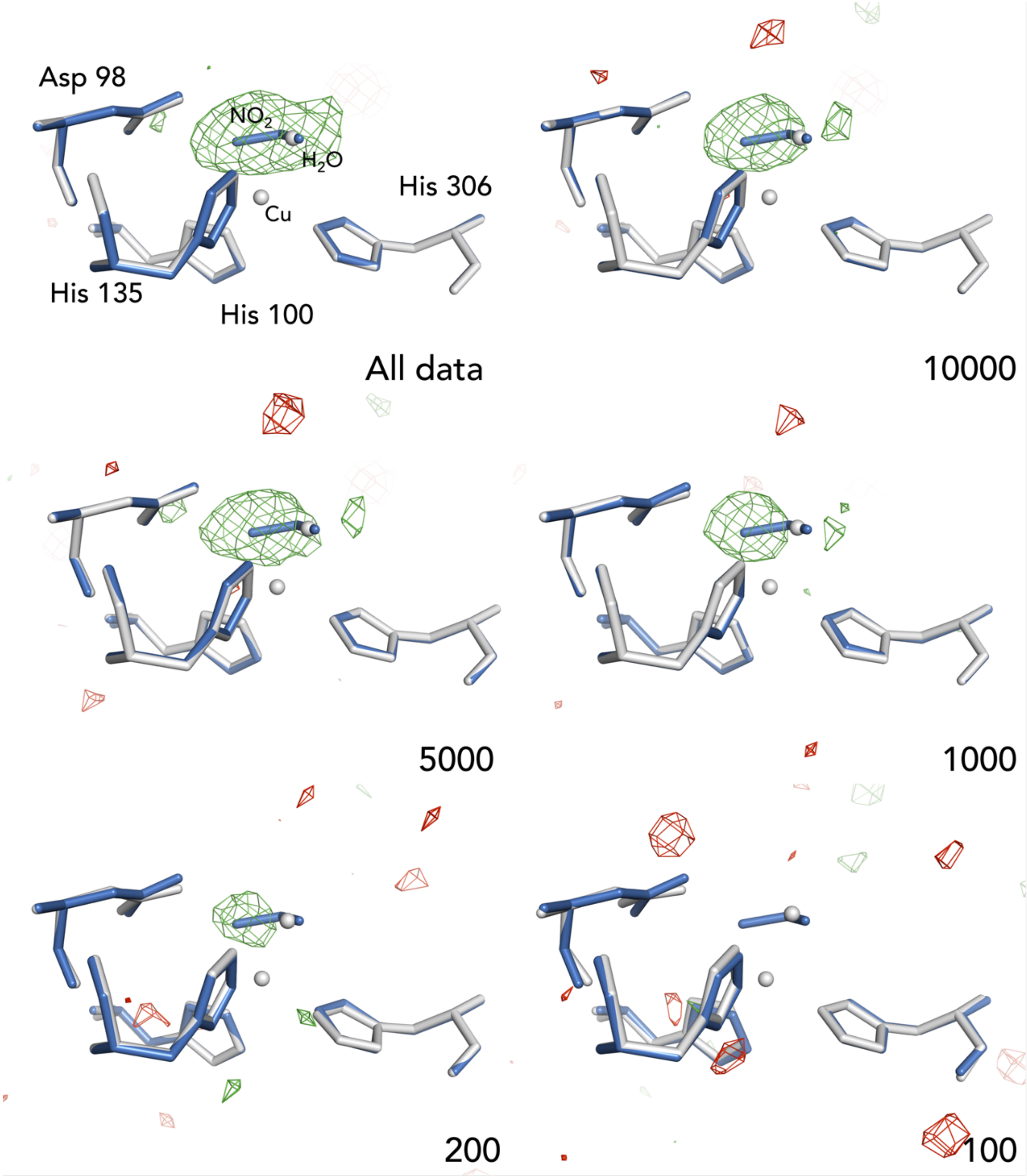

Supplementary Figure S14: *Fo-Fc* omit map, contoured at 3σ from simulated annealing refinement of *Ac*NiR-nitrite subsets versus the SFX structure of native *Ac*NIR with the T2Cu bound water molecule included in the model. The structures from omit refinement are shown in grey, with the superimposed all-data structure of the *Ac*NiR-nitrite complex shown in blue.

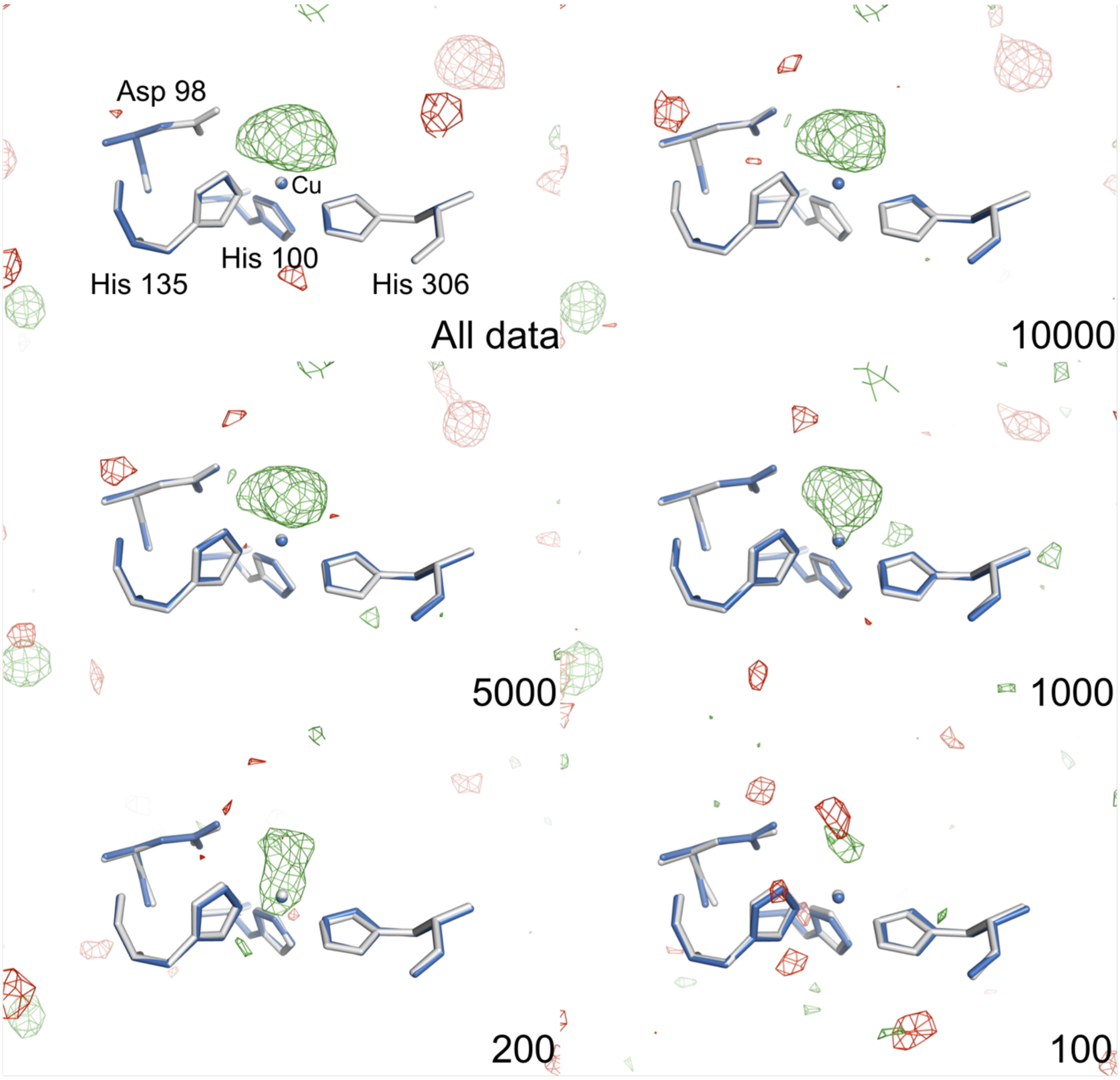

Supplementary Figure S15: *Fo-Fc* omit maps, contoured at 3σ from simulated annealing refinement of *Ac*NiR-nitrite subsets versus the SFX structure of native *Ac*NIR with the T2Cu bound water molecule removed. The structures from omit refinement are shown in grey, with the superimposed all-data structure of the CuNiR-nitrite complex shown in blue.

References

Ebrahim, A., Moreno-Chicano, T., Appleby, M. V., Chaplin, A. K., Beale, J. H., Sherrell, D. A., Duyvesteyn, H. M. E., Owada, S., Tono, K., Sugimoto, H., Strange, R. W., Worrall, J. A. R., Axford, D., Owen, R. L. & Hough, M. A. (2019). *IUCrJ* **6**, 543-551.

Meyder, A., Nittinger, E., Lange, G., Klein, R. & Rarey, M. (2017). *J Chem Inf Model* **57**, 2437-2447.
